# Characterizing particulate polycyclic aromatic hydrocarbon emissions from diesel vehicles using a portable emissions measurement system

**DOI:** 10.1038/s41598-017-09822-w

**Published:** 2017-08-30

**Authors:** Xuan Zheng, Ye Wu, Shaojun Zhang, Jingnan Hu, K. Max Zhang, Zhenhua Li, Liqiang He, Jiming Hao

**Affiliations:** 1 School of Environment, State Key Joint Laboratory of Environment Simulation and Pollution Control, Tsinghua University, Beijing, 100084 P. R. China; 2 State Environmental Protection Key Laboratory of Sources and Control of Air Pollution Complex, Beijing, 100084 P. R. China; 3 Sibley School of Mechanical and Aerospace Engineering, Cornell University, Ithaca, New York, 14853 USA; 4 State Environmental Protection Key Laboratory of Vehicle Emission Control and Simulation, Chinese Research Academy of Environmental Sciences, Beijing, 100012 China

## Abstract

Particulate polycyclic aromatic hydrocarbons (p-PAHs) emitted from diesel vehicles are of concern because of their significant health impacts. Laboratory tests, road tunnel and roadside experiments have been conducted to measure p-PAH emissions. While providing valuable information, these methods have limited capabilities of characterizing p-PAH emissions either from individual vehicles or under real-world conditions. We employed a portable emissions measurement (PEMS) to measure real-world emission factors of priority p-PAHs for diesel vehicles representative of an array of emission control technologies. The results indicated over 80% reduction in p-PAH emission factors comparing the China V and China II emission standard groups (113 μg kg^−1^ vs. 733 μg kg^−1^). The toxicity abatement in terms of Benzo[a]pyrene equivalent emissions was substantial because of the large reductions in highly toxic components. By assessing real traffic conditions, the p-PAH emission factors on freeways were lower than on local roads by 52% ± 24%. A significant correlation (R^2^~0.85) between the p-PAH and black carbon emissions was identified with a mass ratio of approximately 1/2000. A literature review indicated that diesel p-PAH emission factors varied widely by engine technology, measurement methods and conditions, and the molecular diagnostic ratio method for source apportionment should be used with great caution.

## Introduction

Increasing evidence has been reported showing strong associations between vehicle emissions and adverse health impacts^1–7^. Notably, the International Agency for Research on Cancer (IARC), part of the World Health Organization (WHO), has upgraded the carcinogenicity of diesel emissions from Group 2 A (*probably carcinogenic*) to Group 1 (*carcinogenic with sufficient* evidence)^3^. The IARC has concluded that diesel emissions may induce lung cancer and be associated with an increased risk of bladder cancer. Several governmental agencies in the U.S. (e.g., the National Toxicology Program, NTP; Environmental Protection Agency, EPA; and National Institute for Occupational Safety and Health, NIOSH) have also noted that diesel emissions are potentially carcinogenic based on laboratory experiments and epidemiological studies^4–6^. One leading expert in the IARC working group has further highlighted the additional health impacts caused by diesel particulate matter (DPM), which is a complex mixture of carcinogenic chemicals such as polycyclic aromatic hydrocarbons (PAHs)^7^. Diesel emissions of PAHs, including both gaseous and particulate components, comprise a wide spectrum of organic compounds, among which 16 PAH compounds have been classified by the U.S. EPA (see Supplementary Table S1) as priority pollutants (i.e., priority PAHs) because of various toxicological concerns^8–11^.

Particulate PAH (p-PAH) from diesel vehicular exhaust, present in respirable size ranges^12^, in urban areas are of particular concern because of their higher intake fraction than other diesel emission sectors. As an additional research motivation, several PAH species may serve as organic markers to support source apportionment^13^. Previous measurements of p-PAHs emitted from vehicles have primarily conducted in laboratory dynamometer facilities^10, 11, 14, 15^ or through ambient sampling in typical traffic environments (e.g., tunnels and roadsides)^16–18^. These testing methods have a number of useful features but must overcome several limitations. Dynamometer tests are usually conducted according to predetermined cycles that may be simplified (e.g., idling or steady cycles) and may greatly differ from real-world driving conditions^19^. Roadside or tunnel measurements of p-PAHs only represent the fleet-mixture emissions characteristics and lack resolution at the level of individual vehicles. Furthermore, the representativeness of the testing location is often criticized, since these ambient sampling methods usually cover limited traffic circumstances (e.g., in terms of the location, slope, and traffic conditions)^20^.

Increasing attention from both researchers and policy-makers has been focused on the portable emissions measurement system (PEMS), which is an effective tool for evaluating off-cycle emissions (e.g., as in the Volkswagen diesel emission scandal), over the past decade^21–23^. The measurement instrumentation and protocols for the major gaseous pollutants (e.g., CO_2_, CO, HC, and NO_X_) and the particle mass are considered mature, and voluntary or mandatory testing rules have been developed by environmental protection agencies in the U.S. and Europe^24, 25^. Regarding key aerosol species, researchers from Tsinghua University have developed a PEMS method for measuring real-world black carbon (BC) emissions^26, 27^. But for organic aerosol species, a recent study measured on-road emission factors of PAH from diesel vehicles but not discussed the health and environmental implications (e.g., toxicity, source apportionment)^28^. To further explore the diesel vehicle toxic emissions and the emission differences between the PEMS method and previous method (e.g. dynamometer, tunnel and roadside samples), we employed a PEMS system to collect real-world particle samples from diesel vehicles and characterize p-PAH emissions by gas chromatography-mass spectrometry (GC-MS). Fourteen in-use heavy-duty diesel vehicles (HDDVs), a reasonable sample size for a PEMS testing study, were recruited to measure the species-resolved p-PAH emissions under real-world driving conditions. These HDDVs were declared to comply with China I to China V standards and supposed to use improved engine and after-treatment technologies to meet the increasingly stringent emission limits (see Supplementary Table S2). The p-PAH emissions results are presented according to the p-PAH compound, engine type, emission standard category, and traffic conditions. Additionally, a comparison with previous results, the toxic emissions with uncertainty analysis, correlations between real-world BC and p-PAH emissions and implications for aerosol source apportionment are discussed in this article. This study provides useful results for better characterizing real-world p-PAH emissions from diesel vehicles.

## Results

### p-PAHs emission factors

The detailed emission factor results for each vehicle organized by the PAH compound and road type are reported in Supplementary Table S3. Three and 4-ring p-PAHs accounted for a dominant fraction (95% ± 7%; hereinafter, the standard deviation is estimated based on average fuel-based emission factor results for each vehicle in Supplementary Table S3) of the total measured p-PAH emission factors for all vehicles (see Supplementary Figure S1). This overall distribution pattern of PAH species is similar to previously reported results of diesel vehicle emissions. For example, Liang *et al*. reported that the mass fraction of 3 and 4-ring p-PAH was 91% of the total p-PAH emissions from a diesel generator^29^, and Rogge *et al*. reported that 3 and 4-ring p-PAHs were responsible for 82% of the total p-PAH emissions, on average, from two diesel trucks^10^. In this study, Pyrene (Pyr) was the most abundant p-PAH compound, representing 14% to 39% (average of 27% ± 8%) of the total p-PAH emissions from all the tested HDDVs, followed by Phenanthrene (Phe), Fluoranthene (Flu), Fluorene (Fl), and Acenaphthylene (Acy), in descending order of the mass fraction.

**Figure 1:**
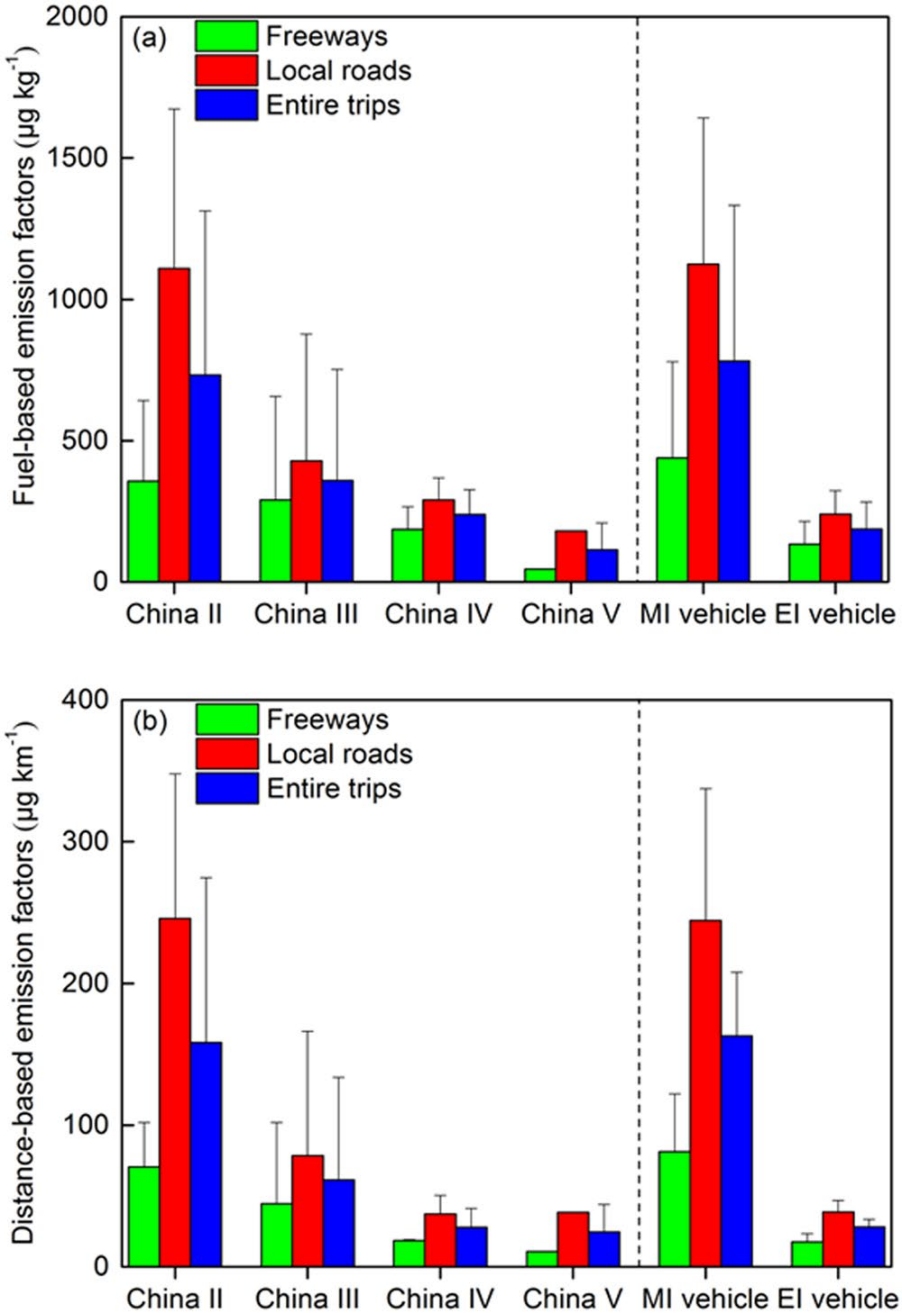
Average p-PAH emission factors for the tested HDDVs according to the emission standard category, engine type and road type, respectively.

In general, the increasingly stringent emission standards functioned to significantly mitigate p-PAH emission factors for the tested HDDVs. As Fig. 1 indicates, the average fuel-based p-PAH emission factors were 733 ± 580 μg kg^−1^, 359 ± 394 μg kg^−1^, and 239 ± 88 μg kg^−1^ (average ± standard deviation, and the values are estimated with average emission factors on local roads and freeways for each vehicle; see Supplementary Table S3b) for the HDDVs that complied with emission standards from China II to China IV (note: hereinafter, we used the mean value of the emission factors for freeways and local roads as the overall results for each vehicle sample and further estimated the group-averaged results according to emission standard category or engine type). These average fuel-based factors are equivalent to distance-based emission factors of 158 ± 116 μg km^−1^, 61 ± 72 μg km^−1^ and 27 ± 14 μg km^−1^, respectively (see detailed emission factors in Supplementary Table S3a). One China V HDDV sample equipped with an electronically-control high-pressure common rail (HPCR) engine had the lowest fuel-based p-PAH emission factor of 113 ± 95 μg kg^−1^ (average ± standard deviation, based on the results on local roads and freeways; see Supplementary Table S3b). This decreasing trend in p-PAH emissions with increasingly stringent emission standards is consistent with the trends in BC and PM_2.5_ emissions^26, 27^. For example, employing a similar PEMS platform, we identified a reduction in average real-world BC emission factors of approximately 80% from China II to China IV^26^.

**Figure 2:**
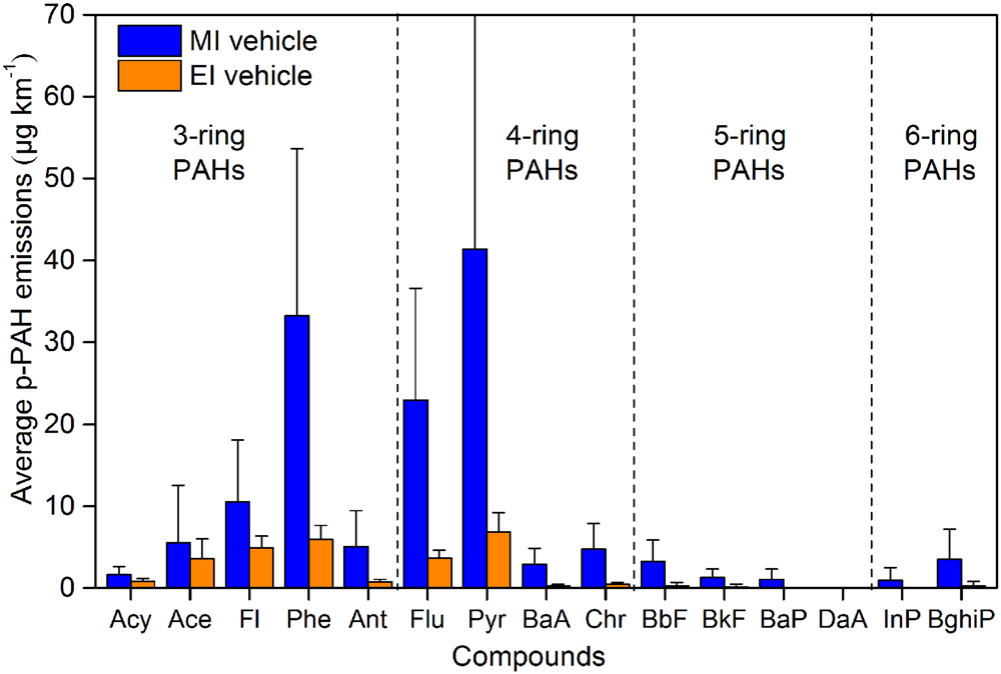
Average distance-based emission factors for each p-PAH component according to the engine type.

As noted previously in Zheng *et al*.^26^, one major reason for the substantial reduction in p-PAH emissions is improved engine technology. The electronically-controlled fuel injection (EI) engines in China III to China V HDDVs can control fuel injection processes more precisely than their mechanical pump fuel injection (MI) engine counterparts (see Supplementary Table S2). Therefore, EI engine could reduce incomplete fuel combustion than MI engine, which is considered as an important cause of p-PAH formation. In general, the EI engine, usually depending on the high-pressure common rail fuel injection technology, raises the combustion pressure, temperature and combustion efficiency in the chamber, which reduces the generation of p-PAH precursors (e.g., C_2_H_2_, C_2_H_4_ and C_3_H_3_)^30^. In this study, the average p-PAH emission factor for the EI engines was 782 ± 378 μg kg^−1^, reduced by 76% compared to that for the MI engines (187 ± 80 μg kg^−1^, see Fig. 1). Extraordinary case involves a China III HDDV with an MI engine (sample #7), which had an overall p-PAH emission factor (1077 μg kg^−1^) that was 6-fold higher than the average of the p-PAH emission factors of the other China III HDDVs with EI engines. The improvement in the fuel injection system of the engine resulted in emissions reductions for all p-PAH species. The species with high abundances (i.e., Pyr, Phe and Flu) accounted for approximately 75% of the total reduction in all p-PAH emissions. Nevertheless, as Fig. 2 indicates, the reductions in p-PAH emissions when comparing the MI engines to the EI engines generally increase with the number of rings or carbon atoms in the p-PAH structure: 61% for 3-ring p-PAHs, 87% for 4-ring p-PAHs and approximately 95% for 5- and 6-ring p-PAHs. Among all PAH species, Benzo[a]pyrene (BaP), Indeno[123 cd]pyrene (InP) and Benzo[ghi]perylene (BghiP) experienced the most significant mitigation effect from engine improvement, resulting in concentrations of these PAHs that were lower than the method detection limits (MDLs) for most of the EI engines. In a previous study, we found that more in-use HDDVs in China declared to comply with China III or even China IV emission standards were actually equipped with MI engines (defined as high emitters), which has resulted from the absence of strict oversight over production conformity^31, 32^. In 2014, the news media reported that numerous counterfeit diesel trucks with improper engine technologies or without the required after-treatment devices were penetrating the market^33^. Therefore, effective inspection programs for the production conformity of HDDVs are needed to prevent the spread of fraudulent products in China and to guarantee the efficacy of stringent standards, which are also in place to protect public health. In addition, since the DPF may exert varying levels of control over soot and organic aerosols, it will be useful to employ the PEMS method to measure DPF-equipped HDDVs when they are readily available (e.g., future China VI HDDVs)^34^.

Traffic conditions are another issue of great concern that affects real-world p-PAH emissions, as the engine load and combustion temperature transiently change according to the traffic conditions. The average fuel-based p-PAH emissions from all the tested HDDVs on freeways were lower than those on local roads by 52% ± 24% (average ± standard deviation, based on the emission reduction ratio of freeways than that on local roads for each vehicle; see Supplementary Table S3). Similar trends in real-world BC and PM_2.5_ emissions with varying traffic conditions have also been observed using the PEMS method^26, 27^. The variation caused by real-world traffic conditions should be considered for different types of diesel fleets (e.g., urban transit buses vs. freight trucks) when estimating p-PAH emissions and their health impacts. Previous laboratory tests have usually applied various testing cycles to reflect distinctive driving conditions. For example, Shah *et al*. reported that the p-PAH emission factors of 11 HDDVs in a high-speed cruise phase (65 km h^−1^) were reduced by 91% compared to those in a transient phase (25 km h^−1^)^35^. In most cases, the detected p-PAH emissions on freeways are lower than those on local roads, although various levels of reduction may occur according to the p-PAH species and engine type. For example, the average fuel-based emission factors of 4-ring PAH species on freeways were lower than those on local roads by 27% (n = 14) (see Supplementary Figure S2). By contrast, the average reduction for 5-ring PAH species, which have higher toxicity factors, reached approximately 60% (n = 7) (see Supplementary Figure S2). Thus, the heterogeneous emission changes of different PAHs to real-world traffic conditions would further lead to greater variations in health impacts, which will be illustrated in below.

### BaP equivalent toxic emission factors

Supplementary Figure S3 presents average BaP equivalent toxic emission factors according to the emission standard, road type and the toxicity contribution of each PAH category. Using the toxicity equivalency factor (TEF) values developed by Nisbet and LaGoy^36^ (see Table S7) and without accounting for ND p-PAH components, the average BaP equivalent emission factors were 2610 ± 2825 ng BaP km^−1^, 207 ± 164 ng BaP km^−1^, 59 ± 31 ng BaP km^−1^, and 37 ± 32 ng BaP km^−1^ for the China II to V HDDVs, respectively. Relative to the China II level, the equivalent toxic emission factors for China III, IV and V HDDVs were reduced by 92%, 98% and 99%, respectively (see Supplementary Figure S3a). The relative reductions in the equivalent toxic emission factors were greater than those in the mass emission factors because, as noted above, the improved engine technology controls the emissions of high-molecular-weight p-PAHs, among which some PAHs have higher TEFs, more effectively than the emissions of the low-molecular-weight counterparts.

5 and 6-ring PAHs were not detected in a considerable number of vehicles, especially for vehicles with EI engines. 5 and 6-ring PAHs typically have higher TEFs than 3 and 4-ring PAHs, which indicates substantial uncertainty in the toxic emission factors because of the bias of the emission factors of 5 and 6-ring PAHs. Previous studies have suggested applying values that are half of the MDLs to estimate toxic equivalent emissions^37, 38^. If we applied half values of the MDLs to replace the blanks for the ND species, the average BaP equivalent emission factors were 3755 ± 2950 ng BaP km^−1^, 479 ± 220 ng BaP km^−1^, 315 ± 203 ng BaP km^−1^, and 181 ± 140 ng BaP km^−1^ (see Supplementary Figure S3b). As a result, the estimated BaP equivalent emission factors for China IV and V HDDVs increased by approximately three times compared to the estimates without considering the ND species. When using half of the MDLs, the total fraction of toxicity contributed by 5-ring PAHs was higher than that contributed by 4-ring PAHs. In addition, different TEF values have been suggested in other studies (see Supplementary Table S4)^39–42^, which could lead to wide ranges in the toxic emission factors, such as approximately 2300–6500 ng BaP km^−1^ for China II HDDVs (note: ND species were not included). This range would be more significant for China IV and V HDDVs, from less than 5 ng BaP km^−1^ to nearly 1000 ng BaP km^−1^. For example, the U.S. EPA has suggested a higher TEF for Pyr (e.g., 0.081 vs. 0.001), which dominated the total p-PAH emissions among all species^36, 39^. No matter which set of TEFs was applied, the reductions in the equivalent toxic emission factors were substantial.

### Relationship between real-world BC and p-PAH emissions

**Figure 3:**
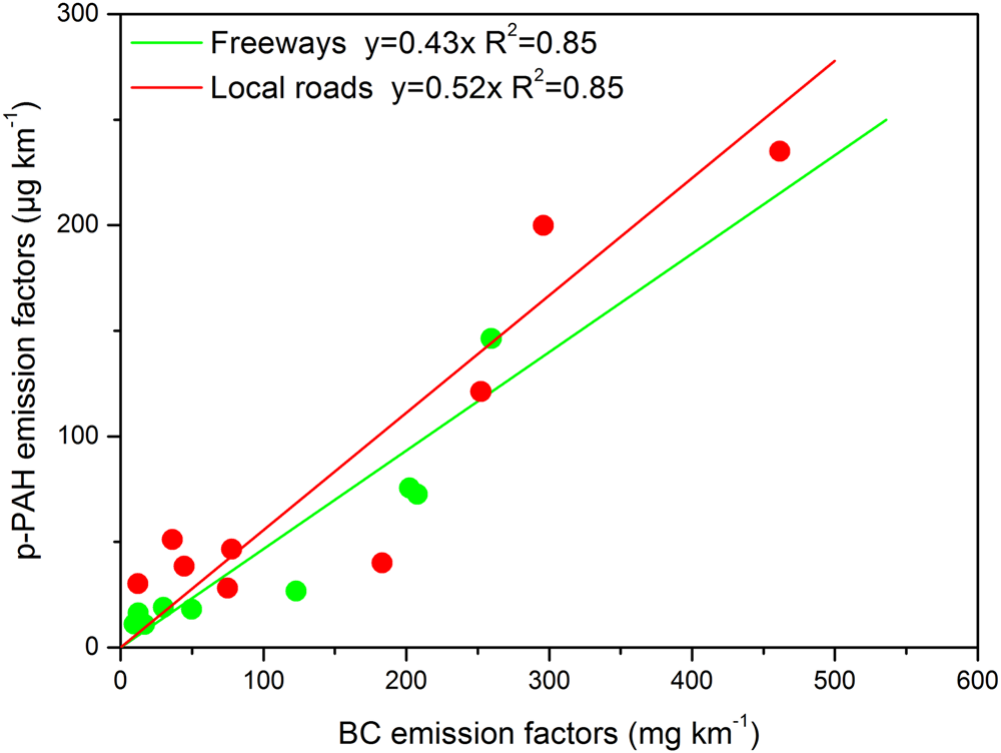
Correlation between p-PAH emission factors and BC emission factors from simultaneous test profiles of nine HDDVs.

Previous studies have used experimental and modeling techniques to discern the growth of PAH molecules to soot during combustion, as well as the strong homogeneity between BC and PAH components in environmental samples^30, 43–45^. Real-world emissions of BC and p-PAHs were jointly measured from nine HDDVs in our study using the PEMS platform. The details of the on-road BC measurements and results were documented by Zheng *et al*.^26^. Figure 3 presents the correlations between the BC and PAH emission factors for the nine HDDVs organized by various road types. In general, the p-PAH emission factors tended to increase with the BC emission factors, with very strong correlations (R^2^~0.85, and t-test p < 0.01). The p-PAH-to-BC mass ratios were rather stable in the diesel vehicle emissions (~1/2000), and the average ratio for freeways (1/2326) was slightly lower than that for local roads (1/1923), which suggest a higher growth tendency to BC from p-PAHs under higher-speed driving conditions (e.g., higher combustion temperatures). These findings may have several useful implications due to the availability of measurement techniques for instantaneous BC emissions. First, BC may act as a reliable indicator of toxicity for DPM emissions together with the species distribution of p-PAHs, which could be further applied in public health studies. Second, modern vehicle emission models have largely applied modal emission rates (e.g., the operating binning method) to simulate complex traffic conditions in the real world, and the instantaneous emission rates for p-PAHs may be developed based on the BC emission rates. Although this PEMS approach could not obtain second-by-second emission rates of p-PAHs to further characterize the instantaneous impacts like driving behaviors (e.g., boost acceleration vs. gentle acceleration), our previously obtained 1-Hz BC emissions profiles may help to understand this issue (see Supplementary Figure S4, originally reported by Zheng *et al*.^26^). For example, the instantaneous BC emission rates of medium-speed and harsh acceleration modes (e.g., Bin 28) would be higher by 2–3 times than those of medium-speed and gentle acceleration modes (e.g., Bin 24)^26^, which would suggest a probably significant effect on p-PAH emissions from various driving behaviors. Third, the relative exhaust-to-ambient phase stability of BC emissions could help to understand the varying phase partitioning of PAHs, because varying p-PAH-to-BC mass ratios between exhaust and ambient samples can be useful to characterize species-resolved transitions of PAHs in the exhaust-to-ambient environment^46^.

## Discussion

### Comparison with previous studies

**Figure 4:**
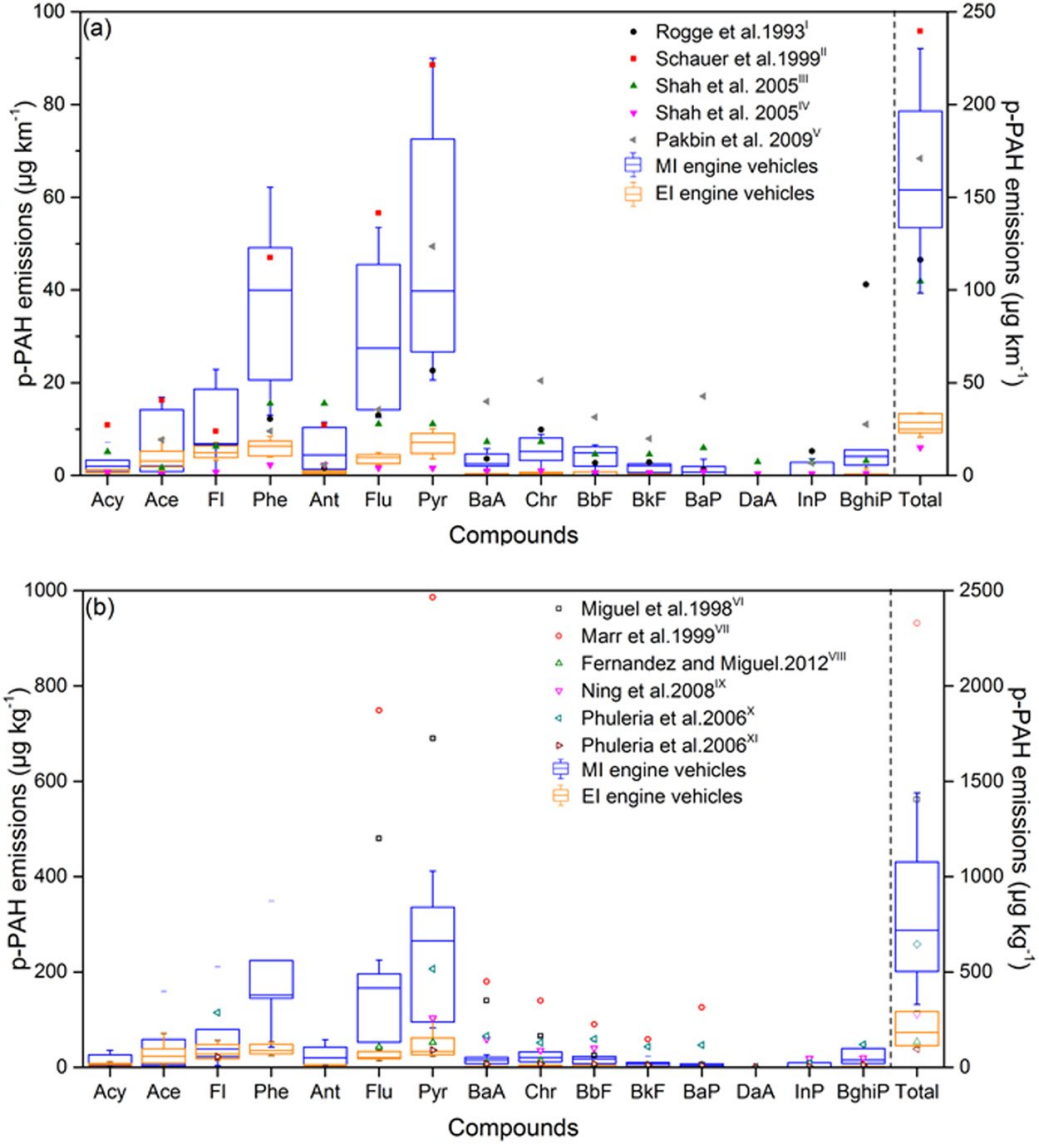
Comparison of the p-PAH emissions from HDDVs determined in this study with those determined in (**a**) dynamometer studies and (**b**) tunnel and roadside studies. Emission factors of each p-PAH compound (left axis) measured in this study are presented in the form of five-number boxplot to reflect inter-vehicle variations, which consists of the minimum, first quartile, median, third quartile, and maximum values. Mean values of emission factors reported in previous studies are marked with the literature sources. Total p-PAHs emission factors (right axis) represent the sum of the fifteen p-PAH compounds detected from each study. Note: (I) dynamometer study of two vehicles with MI engines under steady conditions^10^; (II) dynamometer study of two in-use diesel trucks (model year earlier than 1996) under FTP conditions^11^; (III) dynamometer study of a diesel fleet under transit conditions^14^; (IV) dynamometer study of a diesel fleet under cruising conditions^14^; (V) dynamometer study of vehicles with EI engines under UDDS conditions^15^; (VI) tunnel study at the Caldecott Tunnel^46^; (VII) tunnel study at the Caldecott Tunnel^17^; (VIII) tunnel study at the Caldecott Tunnel^47^; (IX) roadside study near the I-710; (X) tunnel study at the Caldecott Tunnel for ultrafine mode particles^16^; (XI) tunnel study at the Caldecott Tunnel for accumulation mode particles^16^.

Figure 4 presents a species-resolved comparison of the on-road p-PAH emission factors in this study with those determined in previous studies using other measurement methods (e.g., dynamometer, tunnel and roadside samples). In this figure, previous results are indicated with the mean value while the data measured by using PEMS in this study are presented in the form of box plot to represent inter-vehicle emission variations. For example, the MI engine vehicles include 6 China II samples and 1 China III sample, representing a wide range of p-PAH emission factors (e.g., from 83 μg kg^−1^ to 336 μg kg^−1^ for Pyr, see Supplementary Table S3).

Previous dynamometer studies reported measurement results by using distance-based emission factors (see Fig. 4a). Although the specie-resolved emission factors could differ considerable between various studies, the total p-PAH emission factors reported by these dynamometer studies ranged from 105 μg km^−1^ to 240 μg km^−1^ when HDDVs were tested under urban or transit cycles. This range is comparable to the total p-PAH emission level for MI engines measured in this study (i.e., 98 μg km^−1^ to 230 μg km^−1^), because these vehicles tested over dynamometers represented older technology levels with engine model years before 2000. The p-PAH emission factors reported in dynamometer studies are sensitive to the driving cycles applied during testing, as the only exceptional case with a lower total p-PAH emission factor (15 μg km^−1^ under a cruise cycle with less go-and-stop conditions, Shah *et al*., 2005) is also indicated in this figure. Similarly, Pakbin *et al*. measured the emissions from a 1998-model-year diesel truck (Cummins M11 engine, no after-treatment devices) under the UDDS cycle, a transient cycle representing city driving features in the U.S., and determined a total emission factor of 171 μg km^−1^ for 12 detected priority p-PAHs^15^. By contrast, 11 priority p-PAHs were detected by Pakbin *et al*. with a total emission factor of 26 μg km^−1^, after the testing procedure was switched to a steady-speed cruise cycle (80 km h^−1^)^15^. Furthermore, in terms of specie distribution, these dynamometer studies all detected Pyr as the highest emitting priority p-PAH among all detected, which is consistent with our results. Phe and Flu had average emission factors that were higher than or approaching 10 μg km^−1^ in the dynamometer studies and our PEMS study.

Considering all of these results, several implications are relevant to future studies. First, some high-molecular-weight priority p-PAHs (e.g., Dibenzo[ah]anthracene and InP, see Supplementary Table S3) were not detected in previous dynamometer studies or our PEMS samples. Pakbin *et al*. further noted that advanced after-treatment devices for controlling DPM emissions could significantly reduce priority p-PAH emissions by over 99% compared to those from the same engine without an after-treatment device, and no 5 and 6-ring p-PAHs were detected when using these after-treatment devices^15^. A low filter loading and background variability can also be major challenges in measuring particle emissions from ultra-low-emission vehicles with advanced after-treatment devices^14^. Adjusting the dilution factor, increasing the testing duration (e.g., repeated cycles or longer trips), and utilizing multiple filters could help increase the filter loading^14^. Second, Schauer *et al*. revealed that a considerable fraction of detected PAHs were measured in the gas phase under their testing conditions, which would be more significant for low-molecular-weight PAHs (e.g., 3 and 4-ring PAHs)^11^. Nevertheless, this part of the gas-phase PAH emissions would considerably contribute to the primary organic aerosols in the transient exhaust-to-ambient microenvironment.

Different from dynamometer tests, tunnel and roadside studies report fuel-based p-PAH emission factors for entire fleets (see Fig. 4b) because these ambient sampling methods cannot measure the exhaust volume for individual vehicles. Overall, the total p-PAH emission factors from previous tunnel and roadside studies ranged from 95 μg kg^−1^ to 2330 μg kg^−1^, representing a wider variation than that from dynamometer or PEMS studies. A substantial bias of p-PAH emission factors for diesel fleets in these studies can be seen even from measurements at a same tunnel. For example, three papers have reported the p-PAH emissions for diesel fleets at the Caldecott Tunnel in California. Miguel *et al*. and Marr *et al*. both determined p-PAH emission factors for diesel fleets from 1996 to 1997 (1440 ± 160 μg kg^−1^ and 2330 μg kg^−1^) that were more than 10-fold higher than that determined by Fernandez and Miguel for diesel vehicles from 2004 to 2005 (217 ± 109 μg kg^−1^)^17, 46, 47^. For example, the Flu emission factors in Marr *et al*. and Miguel *et al*.’ studies were 749 μg kg^−1^ and 480 μg kg^−1^ which were 5 times and 3 times higher than our results^17, 47^. The large variations between tunnel studies and the gaps compared with PEMS and dynamometer tests mainly attribute to the following reasons. First, as noted above, ambient sampling conditions (e.g., dilution ratio and temperature) are different from the well-controlled conditions in exhaust measurements. Typically, tunnel studies are carried out at lower temperatures and higher dilution ratios, which would result in higher particle-phase fractions of PAHs, compared with PEMS or dynamometer tests^48^. Ambient sampling conditions could vary greatly between various testing campaigns even at a same tunnel. For example, in May *et al*.’s study, the particle-phase fraction of diesel organic emissions was approximately increased from 30% in exhaust chamber to 70% in tunnel after dilution^48^. Eiguren-Fernandez and Miguel^47^ measured the diesel vehicles fleet in Caldecott tunnel in winter and summer, they found that the fleet-averaged p-PAH emission factor for diesel vehicles was 290 μg kg^−1^ in winter, which was approximately double that in summer (140 μg kg^−1^). Second, tunnel studies usually compose all vehicles into gasoline and diesel fleets separately and estimate the fleet-average emission factors based on the carbon balance and multivariable regression analysis method^49^. However, the traffic fraction of diesel vehicles and the background-subtracted concentrations of CO_2_ and CO may also create significant uncertainty in the calculation of the emission factors of p-PAHs for diesel vehicles^50–52^. For instance, Dallmann *et al*. estimated that diesel vehicles accounted for less than 1% of all traffic volume but 45% ± 8% of BC concentrations^50^, which are in strong association with p-PAH emissions. Ježek *et al*. suggested that a change of ±1 standard deviation in the background levels of CO_2_ could change pollutant emission factors by −40% to + 80%^51^. Therefore, there are research needs to develop a PEMS method that can jointly and accurately measure the gas- and particle-phase PAHs emitted from individual vehicles, and further comparatively analyze exhaust and ambient samples to better characterize and simulate the species-resolved dynamics of PAHs in the near-traffic environment^53, 54^.

### Molecular diagnostic ratios of p-PAHs in diesel emissions

Molecular diagnostic ratios (MDRs), the ratios of defined pairs of individual PAH compounds, have been widely applied as organic markers of various anthropogenic sources of PAH emissions^55^. These organic markers can be further used in conjunction with the chemical mass balance (CMB) method to conduct source apportionment for primary organic aerosols^56, 57^. In previous studies, several MDRs, depending on the priority PAH, have been used to infer the source of diesel vehicle emissions. The MDRs from this and previous studies that have been widely used to infer source characteristics (e.g., Fluoranthene/Pyrene + Fluoranthene, Flu/Pyr + Flu; Anthracene/Phenanthrene + Anthracene, Ant/Phe + Ant; and Benzo[a]anthracene/Chrysene + Benzo[a]anthracene, BaA/Chr + BaA) are listed in Table 1. Our PEMS results indicate that the variations in the MDRs due to engine technology and traffic conditions are not significant. For Flu/Pyr + Flu, the overall ratio in this study was 0.40 ± 0.03, which is within the range of the ratios determined in previous studies and close to the values reported by Schauer *et al*. and Rogge *et al*. determined using a dynamometer and in previous tunnel or roadside studies^10, 11^. The MDRs of Flu/Pyr + Flu in this study are not consistent with Ravindra *et al*.’s recommendation that 0.5 is a breaking point for Flu/Pyr + Flu that can be used to distinguish diesel and gasoline combustion sources (e.g., Flu/Pyr + Flu > 0.5 for diesel and Flu/Pyr + Flu < 0.5 for gasoline)^58^. Katsoyiannis *et al*. suggested that Flu/Pyr + Flu > 0.4 represents pyrogenic sources (e.g., fuel combustion), which may also have substantial uncertainty^55^. Furthermore, our average MDRs of Ant/Phe + Ant and BaA/Chr + BaA are both close to the lower limits for inferring combustion sources suggested by Katsoyiannis *et al*. (0.1 and 0.35)^55^. From Table 1, we can observe a rather wide spectrum of MDRs of Ant/Phe + Ant, from 0.05 to 0.21. Meanwhile, the MDRs of BaA/Chr + BaA from tunnel and roadside studies are significantly higher than those directly derived from exhaust samples. Additional MDRs have been proposed to characterize sources related to diesel vehicle emissions, e.g., BaP/BghiP (>0.6 for traffic emissions; 0.38 ± 0.26 in this study, n = 4), InP/InP + BghiP (0.35 to 0.70 for diesel emissions; 0.34 ± 0.06 in this study, n = 3), Benzo[b]fluoranthene/Benzo[k]fluoranthene (BbF/BkF) (>0.5 for diesel, 2.6 ± 1.6 in this study, n = 14), and Pyr/BaP (~10 for diesel emissions; 53 ± 58 in this study)^55, 58^. The difficulty in detecting medium- and high-molecular-weight p-PAHs in diesel exhaust samples is dependent on the MDRs of 5 and 6-ring p-PAHs (e.g., BaP, InP and BghiP). In addition to the uncertainty in the measurement profiles, heterogeneous gas-particle partitioning among various PAH species^59^ and atmospheric reactions between certain PAH species with oxidants (e.g., ozone and nitrogen oxides) may also introduce bias. Therefore, the efficacy and accuracy of using these suggested characteristic MDRs to apportion diesel combustion sources in an ambient environment may be substantially uncertain. Organic markers for traffic-related emissions should be used with great caution, and similar concerns have been also attained by previous studies^13^.

**Table 1:** Molecular diagnostic ratios (MDRs) to infer source characteristics.

Test method	Sources and conditions	Flu/Pyr + Flu	Ant/Phe + Ant	BaA/Chr + BaA
PEMS (this study)	MI engines on freeways	0.40 ± 0.04	0.12 ± 0.03	0.28 ± 0.07
	MI engines on local roads	0.44 ± 0.13	0.09 ± 0.03	0.34 ± 0.12
	EI engines on freeways	0.37 ± 0.04	0.10 ± 0.02	0.34 ± 0.08
	EI engines on local roads	0.38 ± 0.08	0.10 ± 0.02	0.37 ± 0.08
	Overall	0.40 ± 0.03	0.10 ± 0.02	0.33 ± 0.09
Dynamometers	Rogge *et al*.^10^	0.37	0.12	0.36
	Shah *et al*. (for creep, transit and cruise cycles)^35^	0.28, 0.26 and 0.26	0.07, 0.05 and 0.03	0.53, 0.50, and 0.51
	Riddle *et al*. (PM_1.8_ and PM_0.1_ fractions)^60^	0.31 ± 0.04 and 0.31 ± 0.06	0.05 and 0.25	
	Schauer *et al*. (particle phase only)^11^	0.39	0.19	0.33
	Laroo *et al*. (one 1993 Cummins MI engine and one 2008 Cummins EI engine, no after-treatment devices)^61^	0.64 and 0.31	0.15 and 0.07	0.34 and 0.48
	Pabkin *et al*. (values for the UDDS and cruise cycles respectively)^15^	0.22 and 0.26	0.20 and 0.21	0.44 and 0.41
Tunnels and roadsides	Miguel *et al*. (Caldecott Tunnel, August 1996)^36^	0.41		0.68
	Marr *et al*. (Caldecott Tunnel, August 1997)^17^	0.43		0.56
	Phuleria *et al*. (Caldecott Tunnel, August to September 2004; values for accumulation and ultrafine fractions)^16^	0.38 and 0.36		0.50 and 0.56
	Ning *et al*. (I-710 in Los Angeles, one major road with HDDVs accounting for 20% of total traffic)^18^	0.41		0.57
Characteristic MDRs to infer emissions sources by previous studies	Katsoyiannis *et al*.^55^	<0.4 for petroleum sources, and >0.4 for combustion sources; 0.4~0.5 for fuel combustion, and >0.5 for coal and biomass burning;	<0.1 for petroleum sources, and >0.1 for combustion sources;	<0.2 for petroleum sources, and >0.35 for combustion sources;
	Yunker *et al*.^62^	Petroleum sources: 0.26 ± 0.16 for diesel, 0.22 ± 0.07 for crude oil, 0.46 for kerosene, and 0.29 lubricating oil; Combustion sources: 0.39 ± 0.11 for diesel, 0.44 for gasoline, 0.50 for kerosene, and over 0.5 for coal and biomass burning;		
	Ravindra *et al*.^58^	>0.5 for diesel and <0.5 for gasoline		0.50 for diesel and 073 for gasoline

## Conclusion

We employed a PEMS to collect on-road particle samples from fourteen in-use heavy-duty diesel vehicles to address the concern about potential discrepancy between real-world p-PAH emissions and results measured in laboratory. Specie-resolved emission factors of fifteen priority PAH compounds for each individual vehicle sample. The results indicate that 3 and 4-ring p-PAHs were dominant components (95% ± 7%) of total p-PAH emissions. The average fuel-based p-PAH emission factors are 733 ± 580 μg kg^−1^, 359 ± 394 μg kg^−1^, 239 ± 88 μg kg^−1^ and 113 ± 95 μg kg^−1^for China II to China V heavy-duty diesel vehicles. The decreasing trend in p-PAH emissions suggest that tightened emission standards could effectively mitigate real-world p-PAH emissions from heavy-duty diesel vehicles, as improved engine technologies (electronically-controlled fuel injection engines) would be required to penetrate diesel fleet to replace older engine generations (mechanical pump fuel injection engines). Based on the toxicity equivalency factor values developed by Nisbet and LaGoy (1992), the average BaP equivalent emission factors for detected p-PAH compounds are 2610 ± 2825 ng BaP km^−1^, 207 ± 164 ng BaP km^−1^, 59 ± 31 ng BaP km^−1^, and 37 ± 32 ng BaP km^−1^ for the China II to V heavy-duty diesel vehicles. This is mainly because that the improved engine technology effectively controls the emissions of high-molecular-weight p-PAHs, among which some PAHs have higher toxicity equivalency factors.

Real-world p-PAH emission profiles can improve understand the effect from on-road traffic conditions. The average fuel-based p-PAH emissions from all the tested heavy-duty diesel vehicles on freeways are lower than those on local roads by 52% ± 24%. The joint PEMS measurement results of p-PAH and BC indicate strong correlations between p-PAH and BC emissions on both local roads and freeways. The p-PAH emission factors tended to increase with the BC emission factors for heavy-duty diesel vehicles, with average ratios of 1/1923 for local roads and 1/2326 for freeways, respectively. With the real-world p-PAH emission factors, we also evaluate the efficacy and accuracy of molecular diagnostic ratios method that have been widely used as organic makers for source apportionment. Our results suggest that molecular diagnostic ratios method can differ significantly from various studies, and using PAH compounds as organic markers to characterize pollution understanding should be considered with great caution.

## Methods

### Vehicle samples and on-road testing routes

Fourteen in-use HDDVs, including 13 diesel trucks and 1 diesel transit bus were recruited in our PEMS testing campaign. The detailed specifications of each vehicle are summarized in Supplementary Table S5. It is noted that the sample sizes of previous dynamometer testing studies for characterizing p-PAH emissions were below ten vehicles^10, 11, 14, 35, 63^. Thus, we consider the sample size of this PEMS study is reasonably adequate. These HDDVs covered a wide range of production years (1998 to 2014) and were declared by their manufacturers to comply with emission standards from China II to China V (equivalent to Euro II through Euro V). Thus, these vehicles could represent both older and modern generations of HDDVs in China. China II HDDVs are classified as “yellow-label” vehicles representing high emitters and are scheduled to be completely phased out in China by 2017^32^. Meanwhile, China IV and China V HDDVs are rapidly penetrating the diesel fleet in China and are required to use improved engine technologies (e.g., electronically controlled, high-pressure common rail fuel injection) to reduce DPM emissions and selective catalyst reduction (SCR) systems to control NO_X_ emissions. Importantly, none of the HDDVs was equipped with a diesel particle filter (DPF) since the DPF is not a mandatory requirement for most HDDVs until the China VI stage^32^. We carefully checked the actual vehicle specifications (e.g., engine type and after-treatment devices) and usage conditions (e.g., mileage) before each testing trip. All six China II HDDVs (#1 to #6) and one China III HDDV (#7) were equipped with mechanical pump fuel injection engines (MI engines), which cannot control fuel injection as precisely as electronically controlled fuel injection engines (EI engines). The other seven HDDV samples (#8 to #14) were EI engines. The HDDVs were operated by profession drivers who held specialized driver licenses (e.g., for operating trucks or buses). Before each test trip, they were trained to have known the PEMS measurement procedure and been required to drive according to the real traffic circumstances and avoid unnecessary boost acceleration.

The on-road tests were conducted in Beijing and Macao, China. The testing routes in the two cities both consisted of two road types with distinctive traffic conditions: local roads representing congested traffic conditions and urban freeways representing relatively medium and high-speed traffic conditions (see Supplementary Figure S5). The tested vehicles were cycled 2–3 times on the same route in each city because, after a few trials, we found that the measurement duration needed to last 1 to 2 h to ensure sufficient particle loading in the filters, necessary for robust chemical analysis. Supplementary Table S6 summarizes the average speed and distance (i.e., effective sampling distance) of each trip during which particle sampling was conducted, organized by vehicle sample number and road type. The average distance of all tested vehicles was 17 ± 4 km on local roads and 47 ± 8 km on freeways. Ultra-low sulfur diesel fuels were used for the HDDVs tested both in Beijing and Macao. All the diesel fuels were obtained directly from the same gas station in each city, and the fuel tanks were drained before testing.

### PEMS setup and on-road experiments

**Figure 5:**
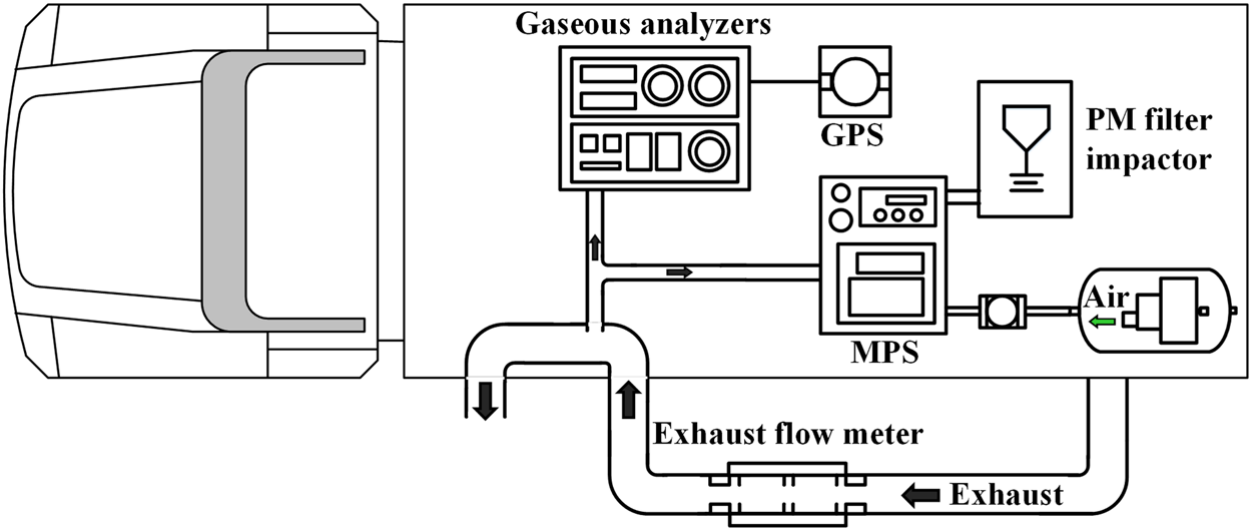
Schematic diagram of the PEMS platform.

The on-board PEMS platform (see Fig. 5) primarily consisted of a SEMTECH ECOSTAR exhaust flow meter (EFM) and gaseous analyzers (Sensor Inc., MI, U.S.), a SEMTECH micro proportional sampling system (MPS; Sensor Inc., MI, U.S.), and a cyclone filter impactor (URG-2000-30FVT; URG Corp., NC, U.S.). The ECOSTAR system is compliant with the in-use emission measurement rule established by the U.S. EPA (CFR40 part 1065)^64^ and comprises a high-speed EFM, a global positioning system (GPS) data logger and gaseous analyzers to measure real-time emissions of CO_2_, CO, THC, and NO_X_. Before each test, the ECOSTAR system was zeroed with pure nitrogen and calibrated using standard gases. Our validation results further indicated that the instantaneous vehicle speeds measured by the GPS data logger agreed very well with the speed data simultaneously derived from an on-board diagnostic (OBD) decoder^26^.

The MPS acted as a proportional diluter and partial-flow sampling system for the DPM samples and was also manufactured to comply with the in-use testing requirements developed by the U.S. EPA. In this study, the inlet volumes of the MPS were maintained at no higher than 5 L min^−1^, and the outlet volumes were maintained at 10 L min^−1^. Thus, the real-time dilution ratios were maintained within the allowed range from 4:1 to 300:1. For the proportional dilution and sampling section, we primarily referred to two indicators of quality assurance and quality control. First, the coefficient of determination (R^2^) of the relationship between the MPS inlet flow volume and the entire exhaust volume measured by the ECOSTAR system for all HDDVs ranged from 0.91 to 0.98, indicating that the MPS did proportionally sample the vehicle exhaust gas (see Supplementary Figure S6). Second, we estimated that the Reynold numbers of the exhaust gases were higher than 24,000, suggesting that the exhaust gases were completely turbulent^26^. In order to verify the exhaust sampling was appropriately proportional (i.e., R^2^ higher than 0.9), we ordered the driver to switch the engine load to make the exhaust flow varying for checking the proportionality. Thus, the tests started with the engines warm.

The URG-2000-30FVT filter impactor was placed in the sampling system, which was heated to 47 ± 5 °C for the entire testing duration. For each vehicle, we used 47 mm quartz fiber filters (Pall Corp., NY, U.S.) to separately collect the DPM samples on freeways and local roads. Prior to use, all the quartz fiber filters were baked in a muffle furnace (550 °C, 5 h)^65^. All filters were sealed in aluminum foil immediately following the completion of the on-road PEMS tests and then stored in a refrigerator at −20 °C for less than 7 days until extraction^66^. We included additional blank samples, which were subjected to the same pre-baking, preservation and chemical analysis procedures but not used for particle sampling, to characterize the background p-PAH levels. The background p-PAH concentrations are listed in Supplementary Table S7.

### Chemical analysis

Before extraction, each filter was spiked with 50 ng of the internal standards (acenaphthylene-d8, phenanthrene-d10, fluoranthene-d10, pyrene-d10, benz[a]anthracene-d10, benzo[a]pyrene-d12 and benzo[ghi]perylene-d12) and extracted in a Soxhlet extractor with 300 ml of a mixture of hexane and dichloromethane (1/1, v/v). The extracts were concentrated by rotary evaporation at 30 °C under vacuum to approximately 1–2 ml, followed by solvent exchange to hexane. Silica gel solid-phase extraction (SPE) cartridges (500 mg, 6 ml^−1^, Agilent Technologies) were employed to clean and fractionate the PAH compounds^66^. The SPE cartridges were eluted three times with 5 ml of a C_6_H_12_-CH_2_C_l2_ mixture (85/15, v/v) at a flow rate of 2 ml min^−1^. The eluate was concentrated to 2 ml by rotary evaporation and dried to 0.5 ml under a gentle stream of nitrogen.

An Agilent 7890 A/5975 C GC-MS system equipped with a DB-5MS column (30 m × 0.25 mm i.d. × 0.25 mm film thickness) was used to analyze the p-PAH contents. 50 ng of benz[a]anthracene-d12 (AccuStandard) was added to the concentrates, of which 1 microliter was then injected into the GC-MS system. The oven temperature program was as follows: 50 °C for 5 min; increased to 200 °C at 19.5 °C min^−1^; increased to 240 °C at 4.5 °C min^−1^; and increased to 290 °C at 2.5 °C min^−1^, followed by a hold of 5 min. Electron impact ionization (EI) was used at 70 eV. Selected ion monitoring (SIM) mode was used for qualitative analysis. The ion source temperature was 250 °C, and the quadrupole temperature was 150 °C. The solvent delay was 4 mins. A series of certified standard mixtures (0.5–125 ng/mL, 15 priority PAHs) were used to quantify the PAH levels. The linear correlation coefficients (R) of the calibration were 0.9945 to 0.9999, and the recovery percentages of the internal standards were 79% to 89%. Finally, 15 U.S. EPA priority PAHs, except for Nap, were analyzed in this study. Nap was not analyzed because of its high volatility and difficult preservation. Notably, some high-molecular-weight priority p-PAHs (e.g., DaA) have never been detected or have been detected at relatively low concentrations in previous studies^10, 11, 46^. Thus, we employed three times the standard deviation of replicate instrumental measurements of the replicate analyses to compute the MDL of each compound, following the U.S. EPA recommended method^67^. The MDL of each PAH is listed in Supplementary Table S8, and concentrations that were lower than MDL are marked as ND (not detected).

### Emissions calculation

For each tested vehicle, the p-PAH emissions were calculated according to the p-PAH species i and road type j on the basis of the testing distance and fuel consumption, as illustrated in Eqs (1) and (2), respectively.

1 $$E{F}_{dis,i,j}=\frac{{M}_{i,j}\times \overline{{R}_{j}}}{{D}_{j}}$$

2 $$E{F}_{fuel,i,j}=\frac{{10}^{3}\times {M}_{i,j}\times \overline{{R}_{j}}\times {w}_{c}}{0.27\times {M}_{C{O}_{2},j}+0.43\times {M}_{CO,j}+0.86\times {M}_{THC,j}}$$

In Eq. (1), $E{F}_{dis,i,j}$ is the distance-based emission factor of p-PAH compound i for road type j in μg km^−1^; ${M}_{i,j}$ is the mass of p-PAH compound i for road type j in μg, analyzed by GC-MS with subtraction of the background concentration; $\overline{{R}_{j}}$ is the average dilution ratio based on the real-time MPS data recorded over road type j; and *D*_*j*_ is the effective testing distance for road type j in km. In Eq. (2), $E{F}_{fuel,i,,j}$ is the fuel-based emission factor of p-PAH compound i for road type j in μg kg^−1^; ${M}_{C{O}_{2},j}$, ${M}_{CO,j}$ and ${M}_{THC,j}$ are the total emissions of CO_2_, CO and THC measured by the ECOSTAR analyzers for road type j in g; and w_c_ represents the mass fraction of carbon in the diesel fuel (0.87 was applied in this study)^68^.

BaP is widely used as a representative PAH with regard to toxicity. In this study, the BaP equivalent toxic emission factor of each vehicle sample was calculated according to the TEF of each PAH compound, as illustrated in Eq. (3).

$$E{F}_{BaP-eq}={10}^{3}\times \sum _{i}E{F}_{dis,i}\times TE{F}_{i}$$

where $E{F}_{BaP-eq}$ is the BaP equivalent toxic emissions in ng-BaP km^−1^ and $TE{F}_{i}$ is the TEF of PAH compound i. The detailed TEFs, which were developed by Nisbet and LaGoy^36^, are listed in Supplementary Table S4. Notably, variable TEFs have been suggested in existing publications, and we later discuss the impact of different TEFs on toxicity characteristics.

## Electronic supplementary material

Supplementary information
