# Supplementary material for: Characterizing particulate polycyclic aromatic hydrocarbon emissions from diesel vehicles using a portable emissions measurement system
Source: Sci Rep. 2017 Aug 30;7:10058. doi: 10.1038/s41598-017-09822-w (PMC5577249; doi:10.1038/s41598-017-09822-w)
Supplement: Supplementary file 1 — Supplementary information [file 41598_2017_9822_MOESM1_ESM.pdf]

Supplementary Information

Characterizing particulate polycyclic aromatic hydrocarbons emissions from diesel vehicles using a portable emissions measurement system

Xuan Zheng [a], Ye Wu [a, b, *], Shaojun Zhang [c, *], Jingnan Hu [d], K. Max Zhang [c], Zhenhua Li [a], Liqiang He [a, d], Jiming Hao [a, b]

[a] School of Environment, State Key Joint Laboratory of Environment Simulation and Pollution Control, Tsinghua University, Beijing 100084, P. R. China

[b] State Environmental Protection Key Laboratory of Sources and Control of Air Pollution Complex, Beijing 100084, P. R. China

[c] Sibley School of Mechanical and Aerospace Engineering, Cornell University, Ithaca, New York 14850, USA

[d] State Environmental Protection Key Laboratory of Vehicle Emission Control and Simulation, Chinese Research Academy of Environmental Sciences, Beijing 100012, China

* Corresponding authors. ywu@tsinghua.edu.cn (YW); sz262@cornell.edu (SZ)

Summary of Supporting Information:

8 supplementary tables (pages S2 to S9) and 6 supplementary figures (pages S10 to S19).

20 pages including the cover sheet.

Supplementary Tables

Table S1. The 16 priority PAHs listed by U.S. Environmental Protection Agency (EPA)

Category	Compound and abbreviation	Molecular formula	Toxicity equivalency factor [a]	Structure
2-ring PAH	Naphthalene (Nap)	$C_{10}H_8$	0.001	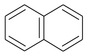
	Acenaphthylene (Acy)	$C_{12}H_8$	0.001	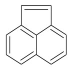
	Acenaphthene (Ace)	$C_{12}H_{10}$	0.001	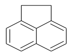
3-ring PAHs	Fluorene (Fl)	$C_{13}H_{10}$	0.001	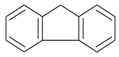
	Phenanthrene (Phe)	$C_{14}H_{10}$	0.001	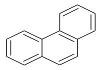
	Anthracene (Ant)	$C_{14}H_{10}$	0.001	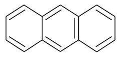
	Fluoranthene (Flu)	$C_{16}H_{10}$	0.001	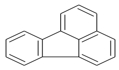
4-ring PAHs	Pyrene (Pyr)	$C_{16}H_{10}$	0.001	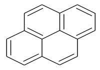
	Benzo[a]anthracene (BaA)	$C_{18}H_{12}$	0.1	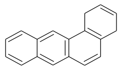
	Chrysene (Chr)	$C_{18}H_{12}$	0.01	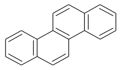
	Benzo[b]fluoranthene (BbF)	$C_{20}H_{12}$	0.1	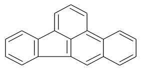
5-ring PAHs	Benzo[k]fluoranthene (BkF)	$C_{20}H_{12}$	0.1	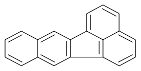
	Benzo[a]pyrene (BaP)	$C_{20}H_{12}$	1	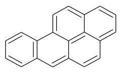
	Dibenzo[ah]anthracene (DaA)	$C_{22}H_{14}$	5	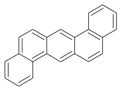
6-ring PAHs	Indeno[123cd]pyrene (InP)	$C_{22}H_{12}$	0.1	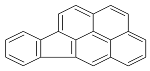
	Benzo[ghi]perylene (BghiP)	$C_{22}H_{12}$	0.001	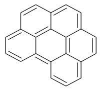

Note: [a] The Toxicity equivalency factors (TEFs) listed in this table were developed by Nisbet and LaGoy, 1992 [S1].

Table S2. Emission regulations of heavy-duty diesel engines in China

Emission standard	Implementation date [a]	Emission limits ($g\ kWh^{-1}$) [b]				Mainstream technologies applied
		CO	HC	NO_X	PM	
China I	2001/09/01	4.5	1.1	8.0	0.36	MI [c] engine
China II	2004/09/01	4.0	1.1	7.0	0.15	MI engine (improved by turbo-charging and inter-cooling system)
China III	2008/01/01	2.1	0.66	5.0	0.10	EI [d] engine
China IV	2015/01/01	1.5	0.46	3.5	0.02	EI HPCR [e] engine, SCR [f, g]
China V	2017/01/01	1.5	0.46	2.0	0.02	EI HPCR engine, SCR [g]

Note: [a] The implementation dates are the requirements for production conformity in nationwide, and the requirements for new model type-approval would be typically earlier by one year. In some developed cities or regions (e.g., Beijing), stringent emission standards would be implemented earlier in public fleets (e.g., transit buses) than the national requirements; [b] Emission limits under steady testing cycles using engine dynamometer; [c] Mechanical pump fuel injection; [d] Electronically-controlled fuel injection; [e] High-pressure common rail; [f] Selective catalyst reduction; [g] SCR is the mainstream option to control NO_X emissions for heavy-duty diesel trucks complying with China IV and China V standards. Diesel particular filters are considered not to be widely used by heavy-duty trucks in China until the future China VI standard is implemented.

Table S3 (a). The detailed distance-based p-PAH emissions for each compound, unit in μg km^{-1}

ID	Road type	Ace	Acy	Fl	Phe	Ant	Flu	Pyr	BaA	Chr	BbF	BkF	BaP	DaA	InP	BghiP	Total
1#	FW	2.45	8.46	10.8	16.0	1.60	21.1	32.7	2.55	4.99	3.22	1.44	1.52	ND	0.79	2.08	110
	LR	4.09	19.8	26.5	25.2	2.82	22.0	32.1	2.51	6.89	6.50	2.77	ND	ND	ND	6.17	157
2#	FW	0.13	1.77	3.18	7.09	0.59	11.3	30.4	3.96	7.21	4.38	1.38	0.15	ND	1.48	2.48	75.6
	LR	1.49	6.80	10.5	18.9	2.08	13.8	22.9	5.37	8.99	8.81	3.50	6.86	ND	4.15	7.01	121
3#	FW	0.38	2.71	3.97	16.7	3.01	8.49	22.9	3.75	5.43	1.84	0.52	0.72	ND	ND	2.13	72.6
	LR	3.56	31.0	41.8	63.3	5.79	19.9	18.4	4.03	4.96	2.17	1.90	ND	ND	ND	3.29	200
4#	FW	3.78	1.74	1.68	12.1	0.65	6.37	6.74	0.63	1.64	2.11	0.83	ND	ND	ND	2.66	41.0
	LR	10.5	2.35	0.025	67.5	2.07	101	138	3.46	6.83	7.66	4.52	ND	ND	ND	8.35	352
5#	FW	0.34	0.44	2.20	19.4	2.81	19.5	46.4	1.48	2.44	ND	ND	0.31	ND	ND	0.77	96.1
	LR	1.38	1.23	10.9	63.5	8.19	71.6	134	10.1	15.2	12.3	5.04	3.52	ND	7.12	20.8	364
6#	FW	0.51	1.11	1.30	5.93	1.40	4.11	7.00	1.06	1.17	1.39	0.53	0.73	ND	ND	1.67	27.9
	LR	3.34	2.91	19.6	92.3	21.6	50.9	72.6	3.38	5.30	3.35	0.78	1.30	ND	ND	2.78	281
7#	FW	1.11	0.55	5.54	43.1	7.00	28.1	58.9	0.92	1.18	ND	ND	ND	ND	ND	ND	146
	LR	3.30	0.09	8.23	81.2	13.8	38.7	86.3	0.99	1.43	0.72	0.14	ND	ND	ND	ND	235
8#	FW	1.37	10.1	6.01	2.38	0.17	2.19	3.33	0.43	0.73	ND	ND	ND	ND	ND	ND	26.7
	LR	1.18	5.93	6.77	5.66	0.65	5.94	10.9	0.61	0.90	0.52	0.25	0.18	ND	ND	0.54	40.1
9#	FW	0.49	1.66	3.18	3.97	0.43	1.73	3.59	0.26	0.33	ND	ND	ND	ND	ND	0.72	16.4
	LR	1.63	6.67	11.1	12.9	1.50	5.32	5.99	0.46	1.06	1.60	0.56	ND	ND	ND	2.40	51.1
10#	FW	0.073	0.027	0.0053	3.19	0.43	2.14	4.03	0.27	0.42	0.41	0.07	ND	ND	ND	ND	11.0
	LR	2.49	4.04	7.78	5.73	0.81	2.25	3.09	0.48	0.52	1.49	1.60	ND	ND	ND	ND	30.3
11#	FW	0.62	0.44	3.76	5.29	1.29	3.61	6.78	0.11	0.10	ND	ND	ND	ND	ND	ND	22.0
	LR	0.65	1.49	6.13	8.04	1.14	6.23	11.3	0.12	0.19	ND	ND	ND	ND	ND	ND	35.3
12#	FW	0.14	0.63	0.98	2.30	0.26	2.93	10.4	0.14	0.31	ND	ND	ND	ND	ND	ND	18.1
	LR	1.14	5.62	6.72	6.13	0.58	2.19	5.08	0.17	0.45	ND	ND	ND	ND	ND	ND	28.1
13#	FW	0.38	2.39	2.57	4.63	0.43	2.32	5.64	0.26	0.45	ND	ND	ND	ND	ND	ND	19.1
	LR	0.95	8.07	7.55	8.13	0.68	5.39	14.6	0.52	0.71	ND	ND	ND	ND	ND	ND	46.6
14#	FW	0.071	0.50	0.69	3.23	0.39	2.52	3.28	0.03	0.05	ND	ND	ND	ND	ND	0.09	10.9
	LR	0.74	3.27	5.78	11.7	1.64	6.46	8.44	0.18	0.26	ND	ND	ND	ND	ND	ND	38.4

Table S3 (b). The detailed fuel-based p-PAH emissions for each compound, unit in μg kg^{-1}

ID	Road type	Ace	Acy	Fl	Phe	Ant	Flu	Pyr	BaA	Chr	BbF	BkF	BaP	DaA	InP	BghiP	Total
1#	FW	20.1	69.7	89.2	132	13.2	174	269	21.0	41.1	26.5	11.8	12.5	ND	6.51	17.2	903
	LR	51.4	249	332	317	35.5	276	403	31.5	86.4	81.6	34.8	ND	ND	ND	77.5	1975
2#	FW	0.51	7.12	12.8	28.5	2.37	45.5	122	15.9	29.0	17.6	5.56	0.62	ND	5.96	10.0	304
	LR	4.40	20.0	31.0	55.7	6.12	40.5	67.6	15.8	26.5	26.0	10.3	20.2	ND	12.2	20.6	357
3#	FW	1.72	12.3	18.1	76.2	13.7	38.7	104	17.1	24.7	8.40	2.36	3.26	ND	ND	9.7	330
	LR	12.0	105	141	214	19.6	67.1	62.1	13.6	16.8	7.34	6.42	ND	ND	ND	11.1	676
4#	FW	13.9	6.36	6.14	44.4	2.38	23.3	24.7	2.32	5.99	7.74	3.02	ND	ND	ND	9.7	150
	LR	38.4	8.59	0.09	247	7.58	368	506	12.7	25.0	28.0	16.5	ND	ND	ND	30.6	1288
5#	FW	1.25	1.62	8.06	71.1	10.3	71.3	170	5.41	8.94	ND	ND	1.13	ND	ND	2.82	352
	LR	5.03	4.52	40.0	232	30.0	261.9	489	36.9	55.6	45.1	18.5	12.9	ND	26.07	76.1	1334
6#	FW	1.85	4.07	4.75	21.7	5.12	15.0	25.6	3.88	4.28	5.08	1.92	2.69	ND	ND	6.12	102
	LR	12.2	10.6	71.6	338	79.0	186	266	12.4	19.4	12.3	2.84	4.75	ND	ND	10.2	1025
7#	FW	7.11	3.50	35.3	275	44.7	180	376	5.85	7.51	ND	ND	ND	ND	ND	ND	934
	LR	17.1	0.49	42.7	422	71.6	201	448	5.15	7.45	3.75	0.74	ND	ND	ND	ND	1220
8#	FW	12.6	92.0	55.0	21.7	1.60	20.1	30.5	3.95	6.65	ND	ND	ND	ND	ND	ND	244
	LR	10.0	50.4	57.6	48.2	5.54	50.5	92.9	5.23	7.65	4.44	2.13	1.51	ND	ND	4.63	341
9#	FW	3.61	12.1	23.3	29.1	3.18	12.7	26.3	1.87	2.42	ND	ND	ND	ND	ND	5.27	129
	LR	8.54	34.9	57.9	67.4	7.85	27.8	31.4	2.41	5.55	8.36	2.95	ND	ND	ND	12.6	268
10#	FW	0.05	0.18	0.04	21.5	2.90	14.4	27.1	1.81	2.80	2.76	0.44	ND	ND	ND	ND	74
	LR	15.1	24.5	47.1	34.7	4.88	13.6	18.7	2.90	3.17	9.01	9.68	ND	ND	ND	ND	183
11#	FW	2.28	1.62	13.8	19.4	4.73	13.2	24.8	0.41	0.35	ND	ND	ND	ND	ND	ND	81
	LR	2.37	5.45	22.4	29.4	4.16	22.8	41.4	0.43	0.70	ND	ND	ND	ND	ND	ND	129
12#	FW	1.92	8.47	13.2	31.0	3.54	39.4	139	1.93	4.20	ND	ND	ND	ND	ND	ND	243
	LR	14.0	69.2	82.8	75.5	7.11	27.0	62.6	2.05	5.53	ND	ND	ND	ND	ND	ND	346
13#	FW	2.62	16.4	17.6	31.7	2.92	15.9	38.6	1.79	3.05	ND	ND	ND	ND	ND	ND	131
	LR	4.81	40.8	38.2	41.1	3.44	27.3	74.0	2.63	3.59	ND	ND	ND	ND	ND	ND	236
14#	FW	0.30	2.14	2.94	13.7	1.65	10.7	13.9	0.11	0.22	ND	ND	ND	ND	ND	0.37	46
	LR	3.48	15.4	27.2	54.8	7.73	30.4	39.7	0.86	1.22	ND	ND	ND	ND	ND	ND	181

Table S4. Different toxicity equivalency factors (TEFs) proposed for each priority PAH compound

Compound	Nisbet and LaGoy[S1]	U.S. EPA[S2]	Chu and Chen[S3]	U.S. EPA[S4]
Ace	0.001	Ndd [a]	Ndd	0
Acy	0.001	Ndd	Ndd	0
Fl	0.001	Ndd	Ndd	0
Phe	0.001	Ndd	Ndd	0
Ant	0.001	0.32	Ndd	0
Flu	0.001	Ndd	Ndd	0
Pyr	0.001	0.081	Ndd	0
BaA	0.1	0.145	0.013	0.1
Chr	0.01	0.0044	0.001	0.001
BbF	0.1	0.14	0.08	0.1
BkF	0.1	0.066	0.004	0.01
BaP	1	1	1	1
DaA	5	1.1	0.69	1
InP	0.1	0.232	0.017	0.1
BghiP	0.01	0.022	Ndd	0

Note: [a] not determined, and the TEF is supposed to zero for calculating the equivalent toxic emission factors in Figure S3.

Table S5. Summary of vehicle specifications

Type	Vehicle ID	Manufacturer	Model year	Emission standard	GVW[a] (t)	Engine category	After-treatment device	Mileage traveled (1000 km)	Engine power rating (kw)	Test time	Test place	With joint testing of BC emissions
Truck	#1	FAW	2003	China II	15	MI [b]		1102	117	2013	Beijing	None
Truck	#2	HINO	1998	China II [c]	26	MI		NA	160	2013	Macao	Yes
Truck	#3	HINO	2004	China II [c]	15	MI		732	180	2013	Macao	Yes
Truck	#4	FAW	2006	China II	20	MI		596	132	2013	Beijing	None
Truck	#5	FAW	2007	China II	25	MI		587	132	2013	Beijing	None
Truck	#6	FAW	2007	China II	24	MI		724	155	2013	Beijing	None
Truck	#7	FAW	2009	China III	24	MI		529	162	2014	Beijing	Yes
Truck	#8	CNHTC	2008	China III	21	EI [d]		NA	210	2014	Beijing	Yes
Truck	#9	FAW	2012	China III	15	EI		263	103	2014	Beijing	Yes
Truck	#10	DCEC	2013	China III	21	EI		219	180	2014	Beijing	Yes
Truck	#11	FOTON	2010	China III	25	EI		178	180	2013	Beijing	None
Truck	#12	DCEC	2014	China IV	14	EI	SCR [e]	3.6	118	2015	Beijing	Yes
Truck	#13	DCEC	2013	China IV	20	EI	SCR	24	180	2015	Beijing	Yes
Bus	#14	King Long	2013	China V	14	EI	SCR	11	165	2013	Macao	Yes

Note: [a] Gross vehicle weight; [b] Mechanical fuel injection system; [c] Approximate to the China II level; [d] Electronically-controlled fuel injection; [e] Selective catalyst reduction.

Table S6. Summary of test routes and average speed by road category for each vehicle tested

ID	Road category	Average speed (km h^{-1})	Distance (km)	ID	Road category	Average speed (km h^{-1})	Distance (km)
#1	FW [a]	44	34	#8	FW	46	34
	LR [b]	24	19		LR	14	11
#2	FW	32	25	#9	FW	54	30
	LR	14	11		LR	19	15
#3	FW	40	31	#10	FW	52	39
	LR	8	6		LR	17	13
#4	FW	52	39	#11	FW	36	27
	LR	22	17		LR	12	9
#5	FW	54	40	#12	FW	55	41
	LR	22	16		LR	18	13
#6	FW	54	40	#13	FW	54	41
	LR	18	14		LR	19	14
#7	FW	44	33	#14	FW	39	30
	LR	17	13		LR	13	9

Note: [a] Freeways (no intersection or traffic signal light); [b] Local roads

Table S7. The background concentrations of PAHs (ng mL^{-1})

Compound	Ace	Acy	Fl	Phe	Ant	Flu	Pyr	BaA
Concentration	5.01±2.16	4.64±2.22	20.67±12.92	9.75±4.00	1.96±0.83	2.84±1.05	2.52±0.37	ND
Compound	Chr	BbF	BkF	BaP	DaA	InP	BghiP	
Concentration	ND	ND	ND	ND	ND	ND	ND	

Table S8. Method detection limitations for specific PAHs (ng mL^{-1})

Compound	Ace	Acy	Fl	Phe	Ant	Flu	Pyr	BaA	Chr	BbF	BkF	BaP	DaA	InP	BghiP
MDL	0.24	0.36	0.45	0.22	0.10	0.30	0.17	0.25	0.32	0.21	0.22	0.20	0.29	0.41	0.51

Supplementary Figures

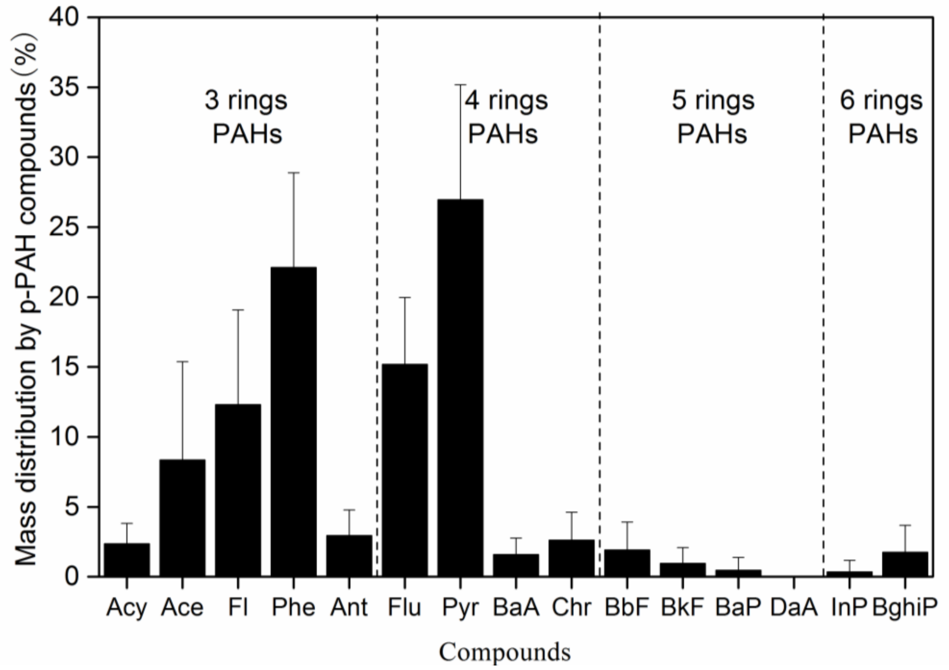

Figure S1. The overall component distribution of p-PAHs based on all samples

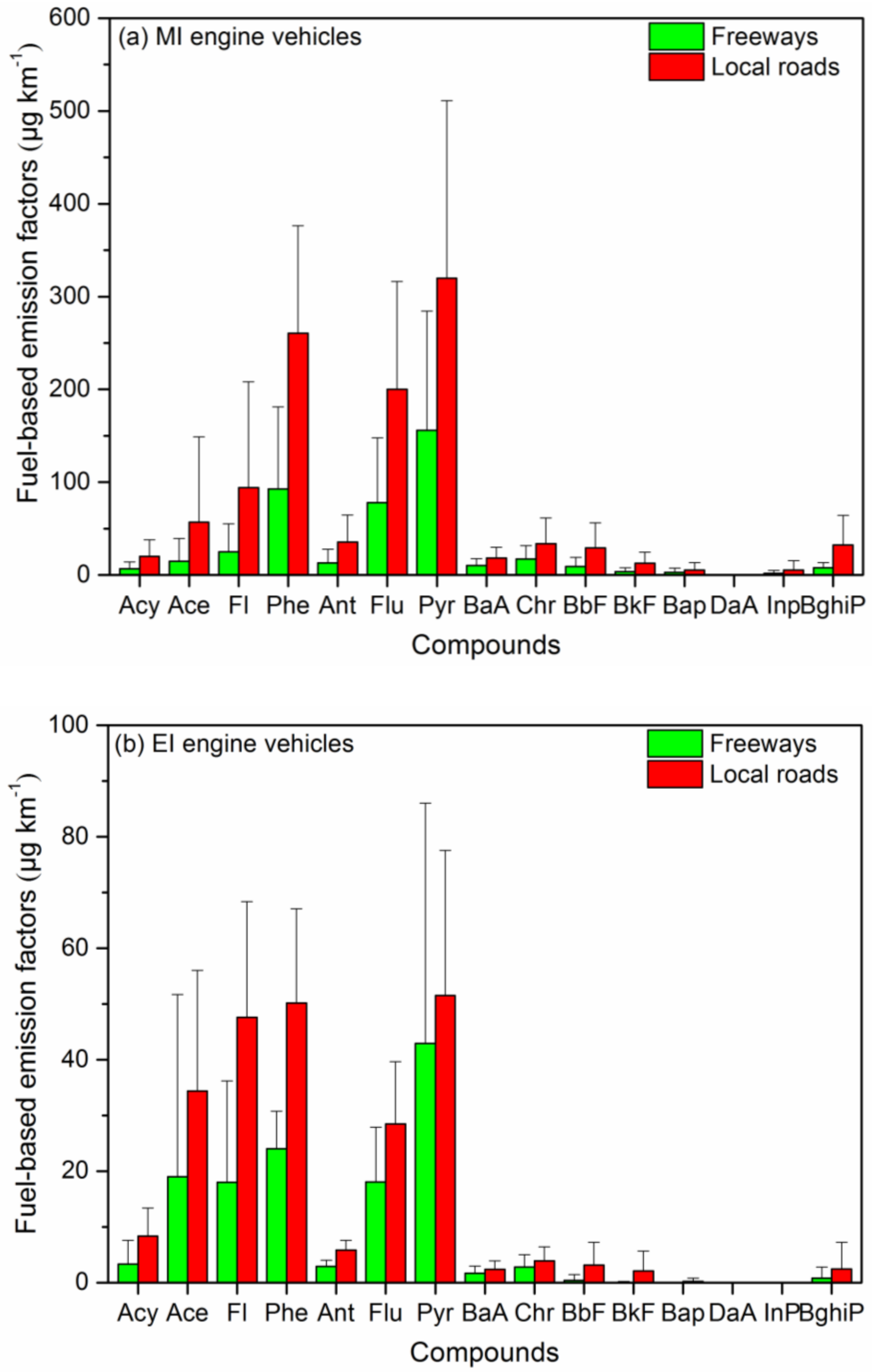

Figure S2. The emission factors of MI and EI engine vehicles on different road types for each compound

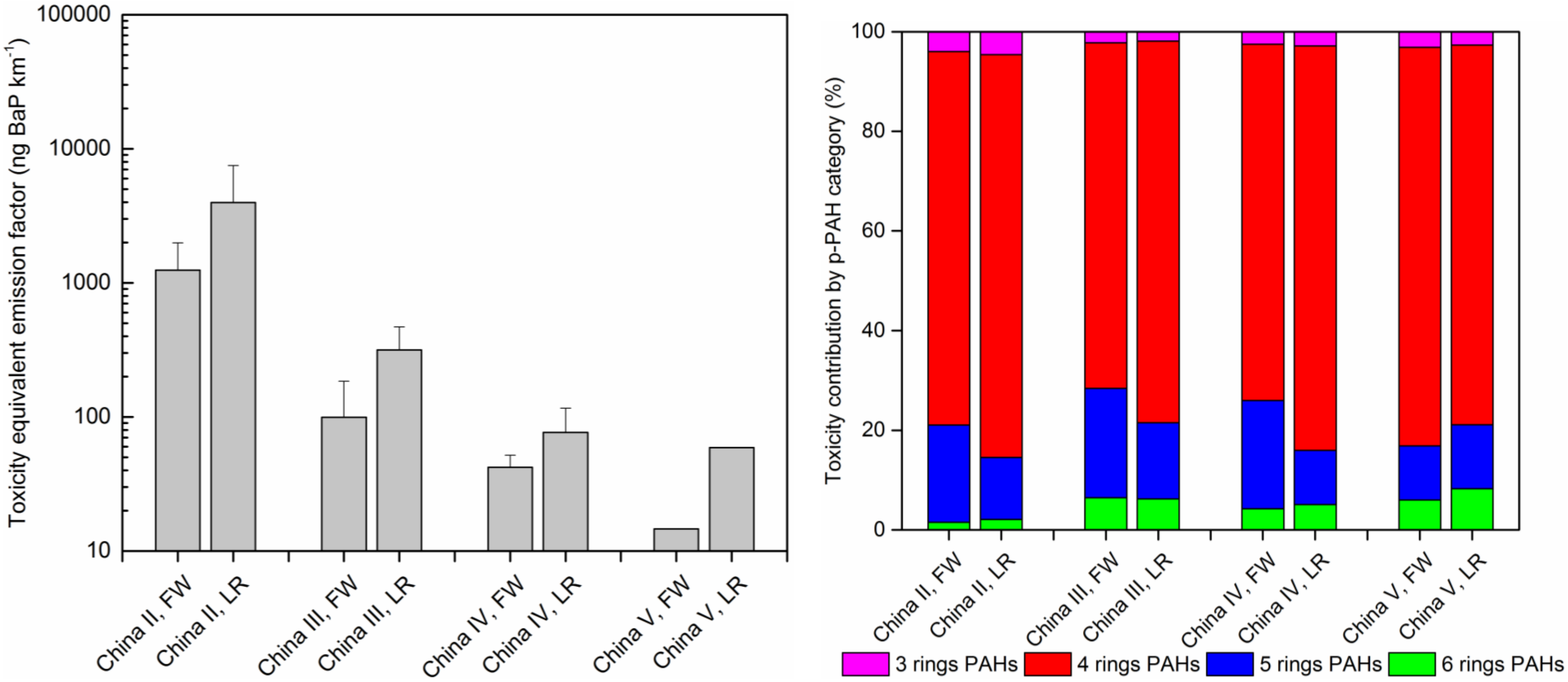

a. TEF values suggested by Nisbet and LaGoy[S1], and emission factors for ND species not accounted

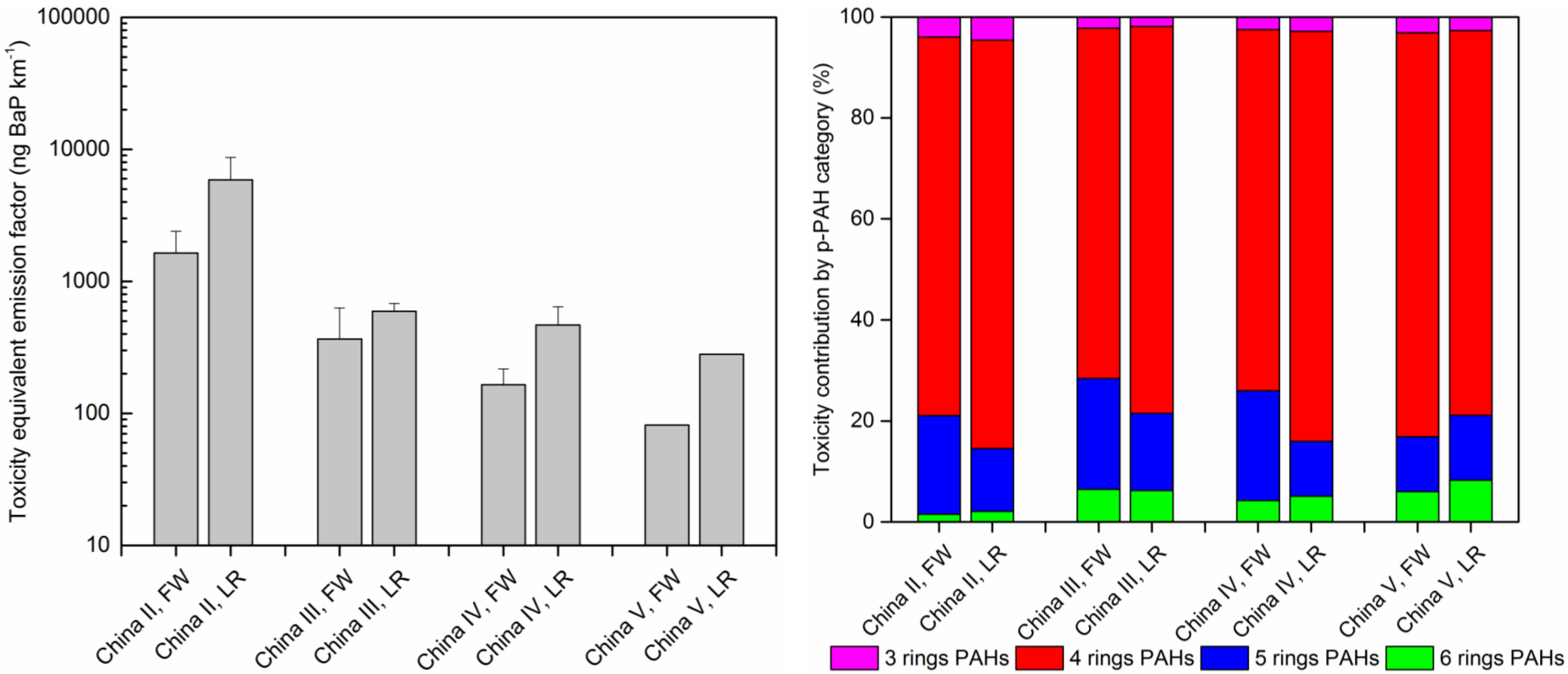

b. TEF values suggested by Nisbet and LaGoy[S1], and half of MDLs to represent emission factors for ND species

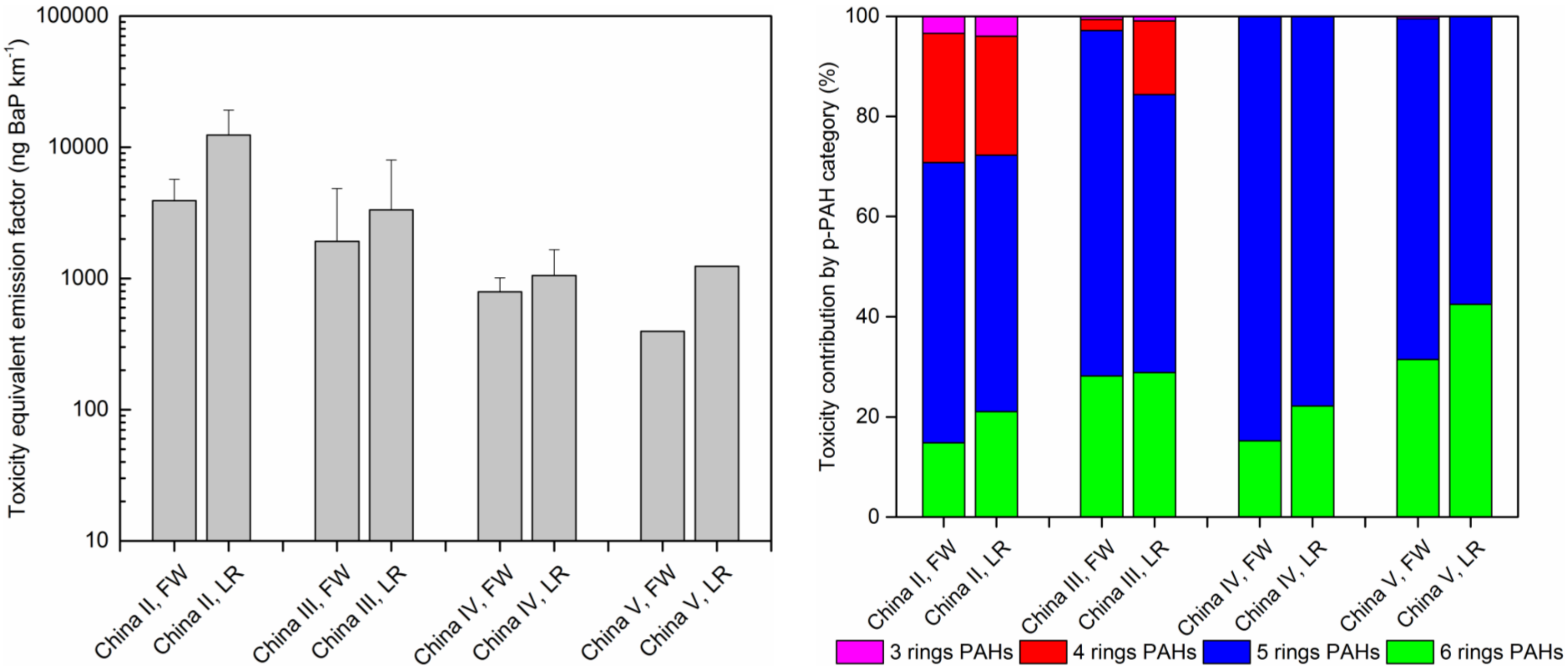

c. TEF values suggested by U.S. EPA[S2], and emission factors for ND species not accounted

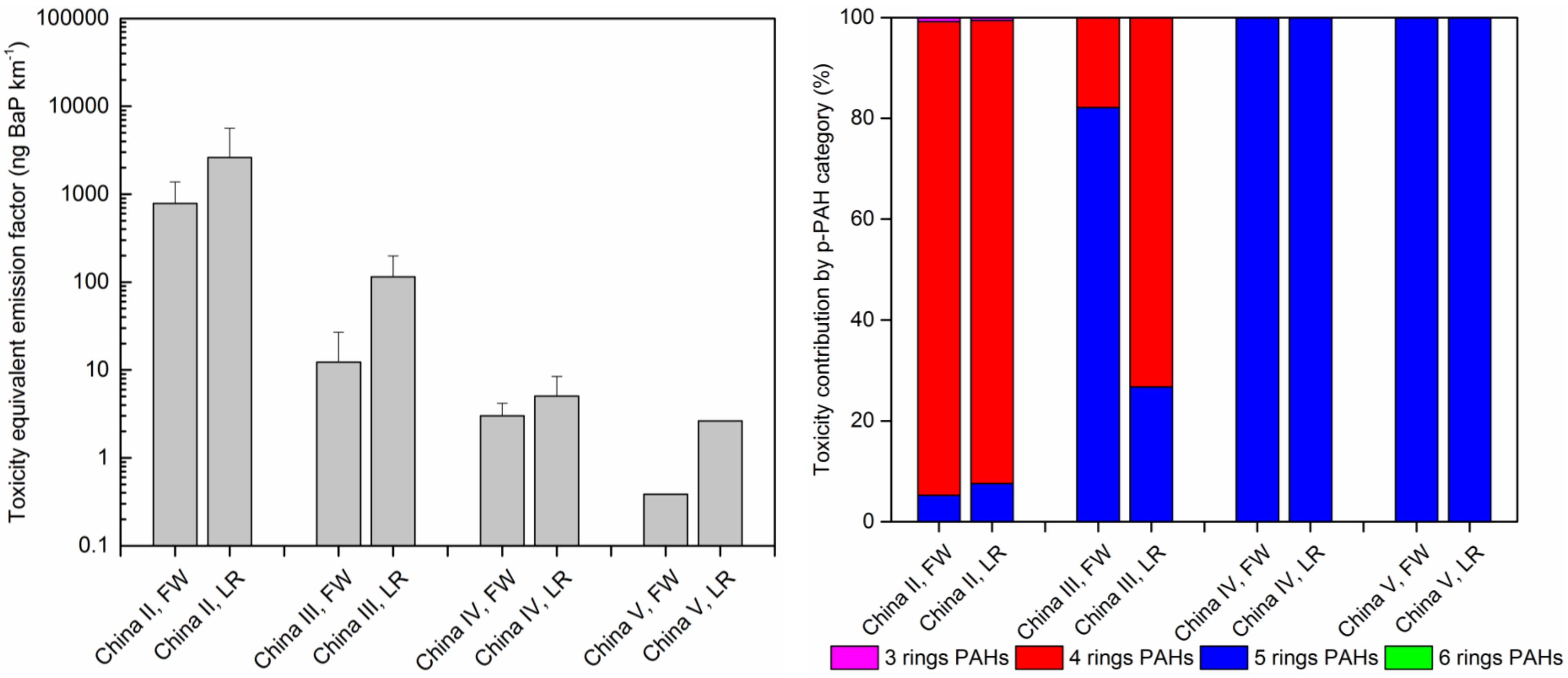

d. TEF values suggested by Chu and Chen[S3], and emission factors for ND species not accounted

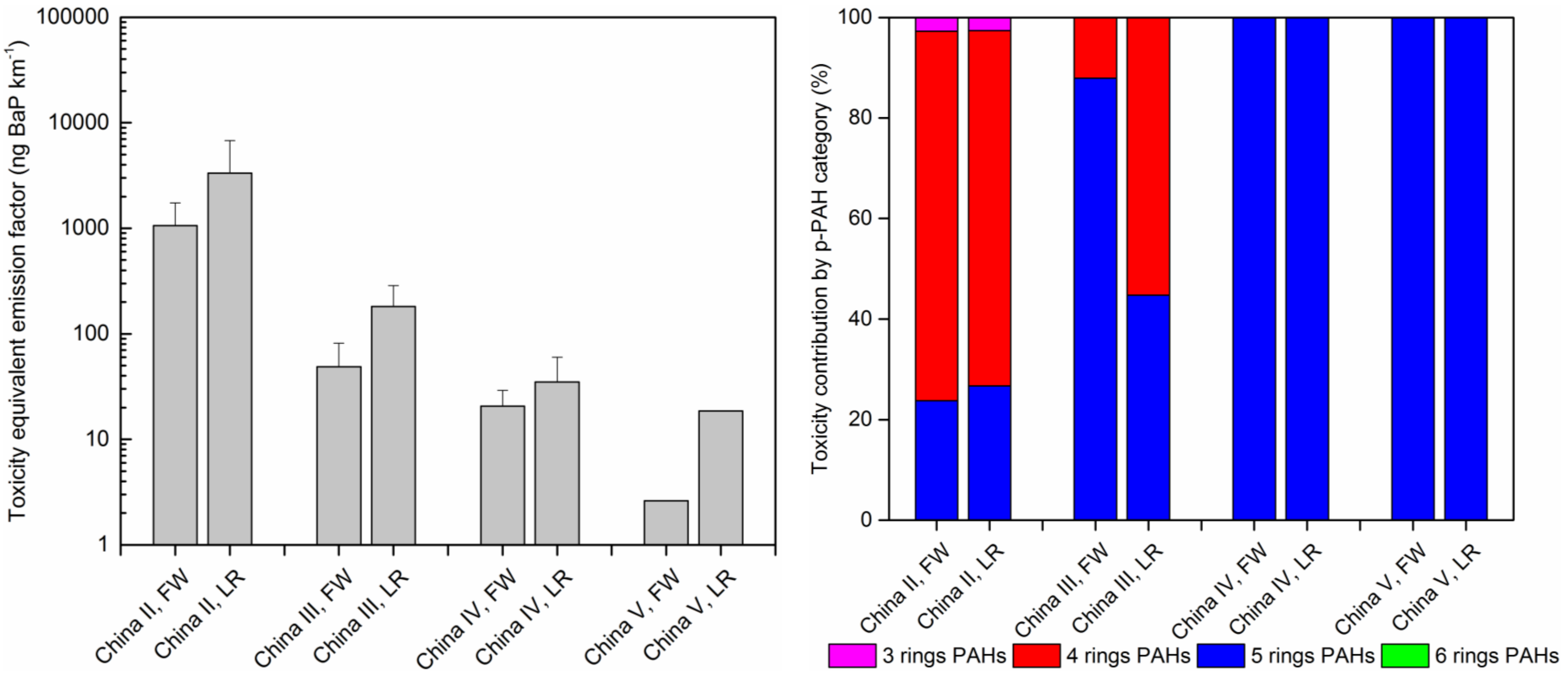

e. TEF values suggested by U.S. EPA[S4], and emission factors for ND species not accounted

Figure S3. The average BaP equivalent emission factors of the tested HDDVs based on different toxicity equivalency factors (TEFs).

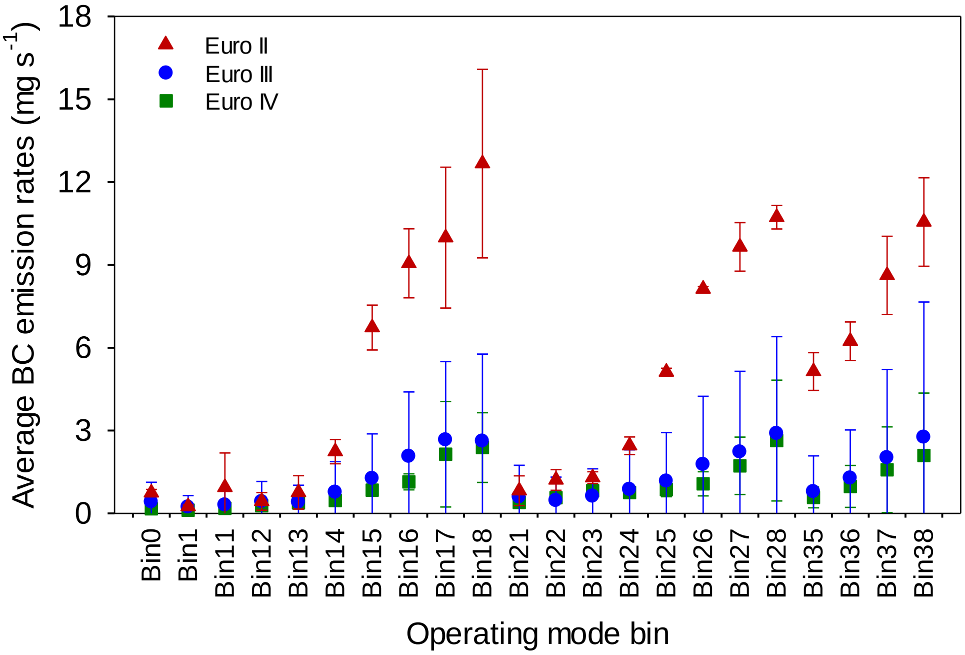

Figure S4. Average BC emission rates for diesel trucks according to operating mode.

Reprinted with permission from Environmental Science & Technology, 49(22), Xuan Zheng, Ye Wu, Jingkun Jiang, Shaojun Zhang, Huan Liu, Shaojie Song, Zhenhua Li, Xiaoxiao Fan, Lixin Fu, Jiming Hao, Characteristics of On-road Diesel Vehicles: Black Carbon Emissions in Chinese Cities Based on Portable Emissions Measurement, Pages No. 13492-13500. Copyright (2015) American Chemical Society.

Note: Definition of operating mode bins by using vehicle specific power and vehicle speed [S5]

VSP (kW t^{-1})	Instantaneous vehicle speed (km h^{-1})			
	v<1.6	1.6≤v<40	40≤v<80	v>80
VSP<-4		Bin 11	Bin 21	
-4≤VSP<2		Bin 12	Bin 22	
-2≤VSP<0		Bin 13	Bin 23	Bin 35
0≤VSP<2	Bin 0	Bin 1	Bin 14	Bin 24	
2≤VSP<4	Deceleration or braking	Idle	Bin 15	Bin 25	
4≤VSP<6		Bin 16	Bin 26	Bin 36
6≤VSP<8		Bin 17	Bin 27	Bin 37
VSP≥8		Bin 18	Bin 28	Bin 38

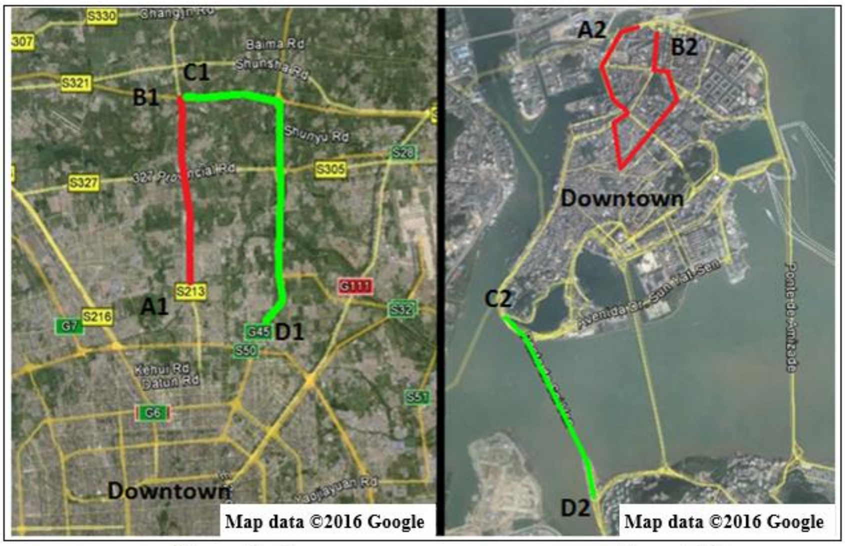

(Panel a) (Panel b)

Figure S5. The vehicle testing routes in Beijing (panel a) and Macao (panel b) (The map source is downloaded freely from Google Map, http://maps.google.com/, testing routes and labels were added using Microsoft Office 2013, https://products.office.com/zh-cn/home?WT.mc_id=oan_winnav_office).

Note: A1-B1: Congested roads in Beijing; C1-D1: Freeways in Beijing; A2-B2: Congested roads in Macao; C2-D2: Freeways in Macao.

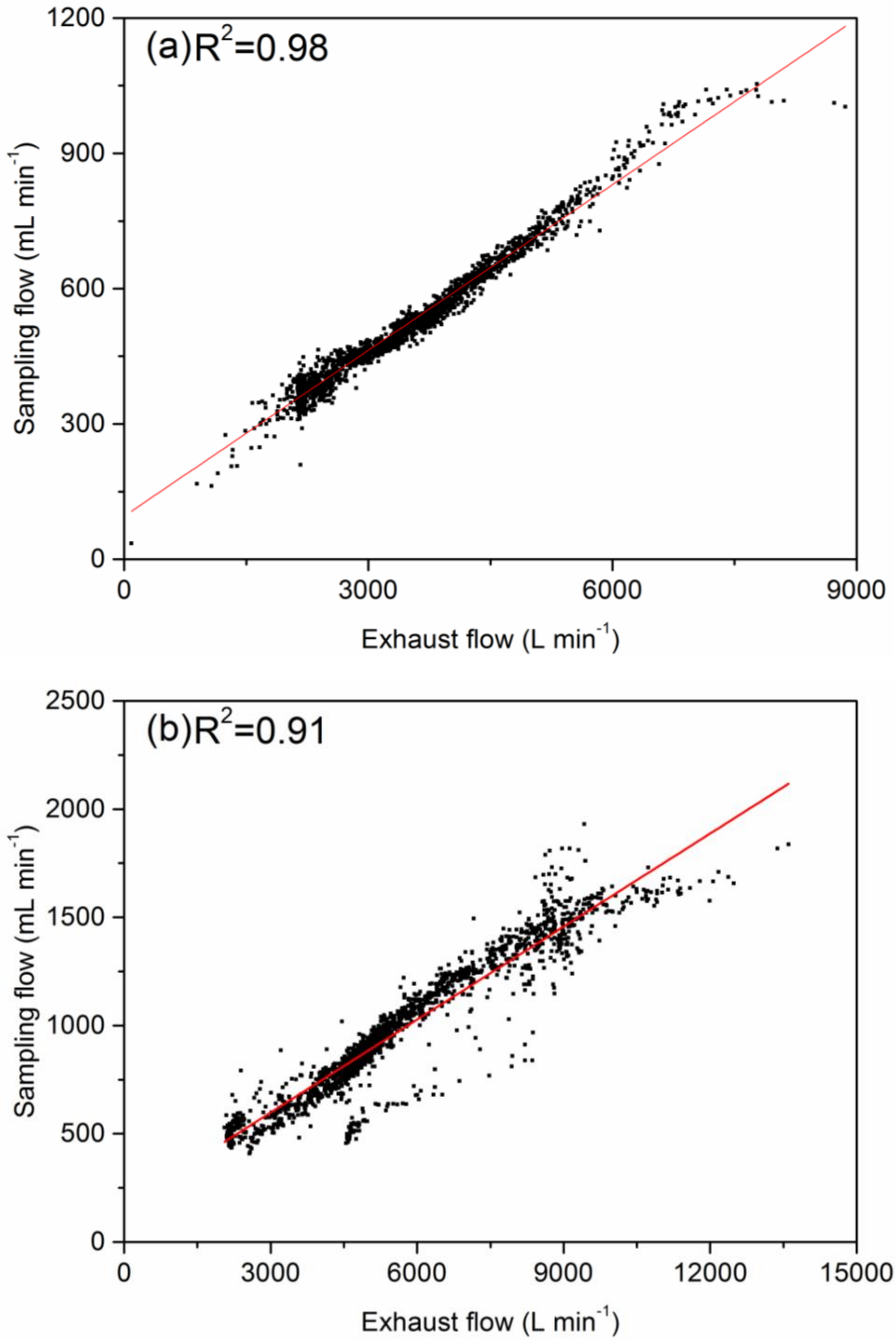

Figure S6. The relationship between sampling flow and exhaust for 10# vehicle (a, the best R square) and 5# vehicle (b, the worst R square).

References

[S1] Nisbet, I.C.T. & LaGoy, P.K.. Toxic equivalency factors (TEFs) for polycyclic aromatic hydrocarbons (PAHs). Regul. Toxicol. Pharm. 16, 290-300 (1992).

[S2] U.S. Environmental Protection Agency (EPA). Comparative potency approach for estimating the cancer risk associated with exposure to mixture of polycyclic aromatic hydrocarbons. EPA600/R-95/108, Washington, DC, U.S. (1988).

[S3] Chu, M.M.L. & Chen, C.W. The evaluation and estimation of potential carcinogenic risks of polynuclear aromatic hydrocarbons (PAH). U.S. Environmental Protection Agency, Office of Health and Environmental Assessment, Office of Research and Development. (1985).

[S4] U.S. Environmental Protection Agency (EPA). Health Effects Assessment of Polycyclic Aromatic Hydrocarbons (PAHs). EPA540/1-86-013, Cincinnati, U.S. (1984).

[S5]. Wu, Y. et al. The challenge to NO_X emission control for heavy-duty diesel vehicles in China. Atmos. Chem. Phys. 12, (19), 9365-9379 (2012).
